# Developmentally Regulated Expression and Activity of Sulphotransferases in the Rat Choroid Plexuses

**DOI:** 10.1111/jnc.70533

**Published:** 2026-07-27

**Authors:** Fiona Qiu, Nathalie Strazielle, Anne Denuziere, Jean‐François Ghersi‐Egea

**Affiliations:** ^1^ Université Lyon‐1, INSERM U1028, CNRS UMR5292 Lyon Neurosciences Research Center, Fluid Team Bron France; ^2^ Brain‐i Lyon France

## Abstract

During development, entry of any substances from the circulation into the brain is tightly regulated by a series of blood–brain interfaces. Notably, the choroid plexuses, which form the blood–cerebrospinal fluid barrier, serve as a key interface for molecular exchange in early life. Control mechanisms within the choroid plexuses include efflux transporters and conjugating enzymes, such as glutathione S‐transferases and UDP‐glucuronosyltransferases, which have been shown to play key roles in safeguarding the developing brain. Sulphotransferases are another family of conjugating enzymes reported to be highly expressed in the choroid plexus in humans and rats during development. However, their activity and functional significance in the central nervous system remain poorly understood. In the present study, sulphotransferase activity was measured in the lateral and fourth ventricle choroid plexus from rats at embryonic Day 19 and postnatal Day (P)1, 3, 8 and 30. Activity was correlated with expression of isoenzymes by RT‐qPCR. Inhibition studies were performed by co‐incubating a prototypical sulphotransferase substrate with a potential substrate or inhibitor. Finally, assays in freshly isolated live tissue were conducted to assess sulphoconjugation under more physiologically relevant conditions. Results showed that both sulphotransferase activity and expression of *Sult1a1* in the choroid plexus were markedly increased at P1 to P3. This distinct temporal pattern suggests age‐ and tissue‐specific roles of choroidal sulphotransferase activity during the early postnatal period. Interactions with xenobiotics and neuroendocrine factors further suggest that these enzymes may contribute to multiple processes during this critical window, including protection against potentially harmful substances and regulation of neurotransmitters. Furthermore, the observed modulation of choroidal sulphotransferase activity by various exogenous substances suggests that developmental exposure could disrupt sulphotransferase‐mediated biological processes, with potential consequences for normal neurodevelopment.

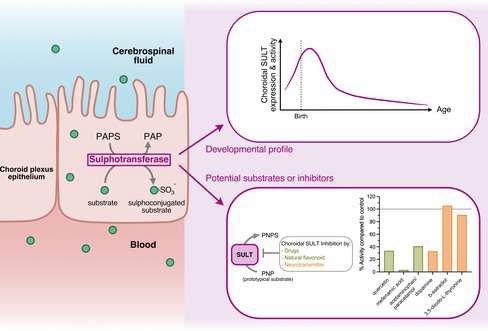

## Graphical abstract

The choroid plexus epithelium forms the blood–cerebrospinal fluid barrier, which plays a key role in regulating molecular exchange between blood circulation and brain. This study demonstrated that both sulphotransferase (SULT) enzymatic activity and the expression of Sult1a1 isoenzyme peaked in the rat choroid plexuses during the neonatal period, suggesting age‐dependent roles for this multi‐specific conjugating enzymatic system at the blood–cerebrospinal fluid barrier during development. Inhibition of SULT activity by certain exogenous and endogenous substances suggested that these enzymes may contribute to multiple biological processes in the choroid plexus, including protection against potentially harmful substances and regulation of neurotransmitters.

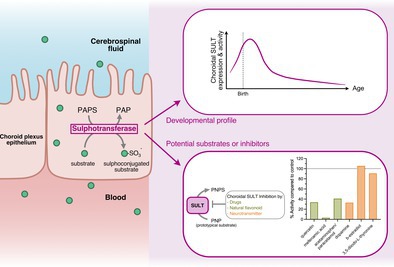

**Abbreviations**
4VCP: fourth ventricle choroid plexus
AC: acetaminophen
BPA: bisphenol‐A
CSF: cerebrospinal fluid
DA: dopamine
E: embryonic day
E2: B‐estradiol
LVCP: lateral ventricle choroid plexus
MFA: mefenamic acid
NAP: 2‐naphthol
P: postnatal day
PAP: adenosine 3′,5′‐diphosphate
PAPS: 3′‐phosphoadenosine‐5′‐phosphosulphate
PNP: p‐nitrophenol
PNPS: p‐nitrophenyl sulphate
QCT: quercetin
RRID: research resource identification
SD: standard deviation
Sult: sulphotransferase
T2: 3,5‐diiodo‐L‐thyronine

## 1 Introduction

**FIGURE 1:**
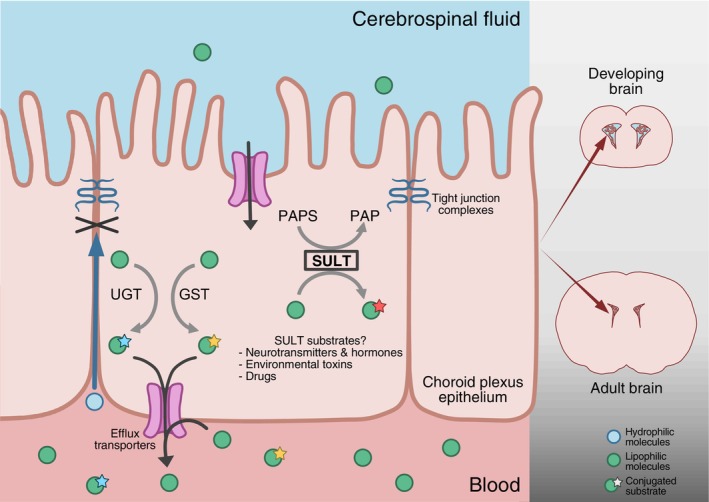
Schematic diagram of the choroid plexus epithelium, focusing on its neuroprotective functions. In the developing brain, choroid plexus represents a prominent interface for exchange between the blood and the cerebrospinal fluid. Tight junction complexes, which restrict the paracellular movement of hydrophilic molecules, are present throughout development. Transcellular entry of numerous lipophilic molecules is restricted by efflux transporters (apical and basolateral) and conjugating enzymes such as glutathione S‐transferases (GST) and UDP‐glucuronosyltransferases (UGT). Sulphotransferases (SULT) are also conjugating enzymes that catalyse the sulphation of their substrates by transfer of a sulphonic group from the universal donor molecule 3′‐phosphoadenosine‐5′‐phosphosulphate (PAPS), which forms adenosine 3′,5′‐diphosphate (PAP). Their developmental expression and activity, as well as substrate specificity, are not well understood.

During development, the internal environment of the highly sensitive brain requires a complex interplay of various biologically active molecules including growth factors, hormones and cytokines, some of which are derived from blood (Mousa and Bakhiet 2013). At the same time, it needs to be protected from potentially harmful substances that may predispose the offspring to neurobehavioural issues, some of which manifest immediately but others do not become apparent until later in life (Giordano and Costa 2012). The entry of any substance from blood into the brain is tightly regulated by a series of interfaces including the blood–brain barrier, located at the cerebral capillary endothelium, and blood‐cerebrospinal fluid (CSF) barrier at the choroid plexus epithelium (Saunders et al. 2016, 2018). The choroid plexuses regulate the composition of CSF by producing or transporting certain nutrients and hormones, and restricting the passage of some harmful substances (Ghersi‐Egea et al. 2018; Lehtinen et al. 2013). While paracellular transfer of water soluble substances into the brain is restricted by junctional protein complexes such as tight junctions, which are present and functional throughout development (Bauer et al. 1993; Ek et al. 2006; Kratzer et al. 2012; Liddelow et al. 2013; Wolburg and Lippoldt 2002), lipophilic molecules may diffuse across the blood–brain barrier and blood‐CSF barrier transcellularly depending on their lipid solubility. Instead, physiological mechanisms such as efflux transporters, found on membranes of barrier‐forming cells, as well as detoxifying enzymes confer a crucial protective role (Ghersi‐Egea et al. 2018; Löscher and Potschka 2005). In particular, the blood‐CSF barrier represents a prominent interface for molecular exchange between the circulation and central nervous system especially in early brain development (Saunders et al. 2023). As illustrated in Figure 1, glutathione S‐transferases (EC 2.5.1.18) in the choroid plexuses are conjugating enzymes that contribute significantly towards overall protection of the developing brain against exposure from harmful compounds especially during the period before the liver reaches full maturity (Ghersi‐Egea et al. 2006; Kratzer et al. 2018). Similarly, the UDP‐glucuronosyltransferases (EC 2.4.1.17) are also a conjugating system that limit brain‐to‐CSF entry of their substrates in rodents (Strazielle and Ghersi‐Egea 1999) (Figure 1).

Brain presence of another class of conjugating enzymes, the sulphotransferases (EC 2.8.2.1), has been reported in humans (Salman et al. 2009; Zhu et al. 1993), and in rodents (Alnouti and Klaassen 2006; Liyou et al. 2003). Sulphotransferases are enzymes catalysing the sulphation of their substrates by transfer of a sulphonic group from the universal donor molecule 3′‐phosphoadenosine‐5′‐phosphosulphate, PAPS (Chapman et al. 2004; Falany 1991, 1997). Different members of the sulphotransferase family interact with a plethora of structurally diverse substrates. Among them, the cytosolic superfamily typically recognises substrates of both endogenous and exogenous origins including steroids, thyroid hormones, catecholamines, therapeutic compounds and environmental toxins (M. W. Coughtrie 2016; James and Ambadapadi 2013). In general, cytosolic sulphotransferases inactivate the biological activity of their endogenous substrates and detoxify xenobiotics (Strott 2002), although in certain situations, sulphoconjugation may result in the production of reactive metabolites including mutagens and carcinogens (Glatt 2000). Sulphoconjugated substances may have reduced permeability across biological membranes not only via increased hydrophilicity, but also by action of ATP‐binding cassette (ABC) efflux transporters that recognise compounds conjugated to a transport motif such as sulphate, glutathione and glucuronate following phase II metabolism (Nowell and Falany 2006; Zhou et al. 2008).

In addition to xenobiotic metabolism, sulphotransferases in the central nervous system are thought to be related to the regulation of neurotransmitters and other neuroendocrine factors (M. Coughtrie 2002). During development, the presence and activity of sulphotransferase have been detected in the fetal human brain (Richard et al. 2001; Wengle 1966). Despite the paucity of information about sulphotransferases in the choroid plexus, available evidence indicated that it appears to be an important site for sulphoconjugation during development. This is supported by studies in human fetuses of 14–20 gestational weeks which showed a strong signal of sulphotransferases in the lateral ventricle choroid plexus by immunoblot, as well as robust enzymatic activity towards p‐nitrophenol, a typical sulphotransferase substrate, at levels approximately 7 times higher than those observed in the cerebral cortex (Richard et al. 2001; Stanley et al. 2005). Similarly, in the developing rat, transcripts of several classes of sulphotransferase were identified in the choroid plexus, and the expression of sulphotransferase *Sult1a1* has been detected from as early as embryonic Day 15 (term is 22 days in rat) (Kratzer et al. 2013) (Figure 1). However, the activity of these enzymes in the choroid plexus, their regional distribution within the central nervous system, as well as their substrates, across fetal and postnatal stages in the rat remains poorly understood. This information could provide some insights into the physiological properties of sulphotransferases at the blood‐CSF barrier during critical periods in development, as well as the extent of protection they offer to the central nervous system. In turn, this may inform how alterations in sulphotransferase activity could contribute to potential adverse effects on brain development.

The present study aimed to establish the developmental profile of sulphotransferase activity in the lateral and fourth ventricle choroid plexus from late gestation fetal (embryonic Day 19) to postnatal Day 1, 3, 8 and 30 rats. Activity was correlated to the choroidal expression of sulphotransferase isoenzymes in age‐matched animals. The extent of inhibition of sulphotransferase activity using various potential inhibitors and/or substrates was determined as a mean to elucidate their possible physiological functions in the developing choroid plexus. Finally, live isolated choroid plexuses were used to show that this tissue has the capacity to synthetise the sulphate donor and generate sulphoconjugates during early development in a physiological cellular environment.

## 2 Methods

### 2.1 Animals & Tissue Collection

Sprague–Dawley rats (RRID: MGI:5651135) used in this study were maintained at the Lyon Neurosciences Research Centre facility. Animal care and procedures were conducted according to the guidelines approved by the French Ethical Committee (decree 87–848) and by the European Community directive 86 to 609‐EEC. It was approved by INSERM institution as part of the accredited FLUID research project, and by IACUC CEEA‐42 (Lyon Ethical committee for animal experimentation in Neuroscience, CELYNE). Animals were kept in a 12‐h light/dark cycle and provided with ad libitum access to standard food and water. Tissue samples were obtained from fetuses at embryonic (E) Day 19 and pups at postnatal (P) Day 1, 3, 8 and 30. Details of animal's experimentations are reported according to the ARRIVE guidelines 2.0 (Percie du Sert et al. 2020). Animals of both sexes were included in this study. Total number of animals used are listed in Table 1.

**TABLE 1:** Number of animals used for different experimental designs.

Age group	Sulphotransferase activity assay	Inhibition assay	RNA extraction	Live choroid plexus assay
E19	34 (3)	—	15 (1)	—
P1	12 (2)	—	12 (4)	14 (3)
P3	11 (3)	47 (5)	8 (2)	—
P8	12 (2)	—	—	—
P30	4 (2)	—	4 (2)	4 (1)

*Note:* Number of litters are indicated in brackets. Both females and males were included at all stages.

Time‐mated primigravida dams at E19 were deeply anaesthetised with inhaled isoflurane (3.5%) and placed on a heated pad for the duration of the experiment. A small abdominal incision was made to expose the uterine horns, and each fetus was exteriorised in sequence. Viability of all fetuses included in the study was confirmed and their sex was visually verified by the presence of testes or uterus. The brains of the fetuses killed by exsanguination and decapitation were removed and placed in cold buffer solution (Hank's balanced saline solution (HBSS)) where lateral and fourth ventricle choroid plexuses were dissected. Postnatal animals were killed by decapitation following inhaled 3.5% isoflurane anaesthesia, and their brains were removed. In E19, P1, P3 and P8 pups, pools of choroid plexuses were formed from 2 to 6 animals of mixed sex. Choroid plexuses from individual P30 male and female animals were used. Samples of the frontal–parietal cortex and liver at all ages as well as placenta at E19 were also collected. All samples were stored at −80°C until use. Tissues used for extraction of RNA were dissected from animals in a similar manner, with additional precautions taken to minimise contamination from RNase. These samples were flash‐frozen in liquid nitrogen before storage at −80°C.

### 2.2 Sulphotransferase Activity Assay in Homogenates of Choroid Plexus and Other Tissues

To assess activities of sulphotransferases in choroid plexus, liver, cerebral cortex and placenta, tissue samples were first mechanically homogenised in a buffer solution (50 mM potassium phosphate, 0.25 M sucrose, 1 mM ethylenediaminetetraacetic acid and 0.1 mM dithiothreitol; pH 7.4) on ice, with appropriate volumes to maintain comparable protein concentrations between age groups. Assay conditions were adapted from an established method (Frame et al. 2000). Each assay mixture (total volume of 250 μL) contained 5 mM p‐nitrophenyl sulphate (PNPS; Sigma‐Aldrich, Cat. No. N3877), 0.5 mM adenosine 3′,5′‐diphosphate (PAP; Sigma‐Aldrich, Cat. No. A5763), 20 μM 3′‐phosphoadenosine‐5′‐phosphosulphate (PAPS; Sigma‐Aldrich, Cat. No. A1651) and 0.1 mM 2‐naphthol (NAP, Sigma‐Aldrich, Cat. No. 70448) in a buffer solution (50 mM potassium phosphate and 5 mM MgCl_2_; pH 6.5). Control assays that omitted 2‐naphthol were included in each run to confirm the absence of its sulphoconjugate. Following incubation at 37°C with agitation in an incubator, reaction was terminated by precipitation of proteins with 250 μL of cold acetonitrile. Incubation time was kept within the linear phase of metabolite formation, which was 1 h for liver samples and 2 h for all other tissues. Samples were then vortexed and centrifuged at 14000 *g* for 10 min at 4°C to obtain supernatant, which was analysed by high‐performance liquid chromatography (HPLC) to quantify p‐nitrophenol (PNP) and 2‐naphthyl‐sulphate as described below. Peak area of 2‐naphthyl‐sulphate was found to be linearly proportional with that of PNP and inversely proportional with the disappearance of 2‐naphthol, therefore its concentration was calculated from the correlation. Following measurement of protein concentrations, enzymatic activity is expressed as nmol/min/mg of protein in homogenate.

### 2.3 mRNA Expression by Quantitative Polymerase Chain Reaction (RT‐qPCR)

Developmental expression of genes related to sulphoconjugation mechanisms was characterised in E19, P1, P3 and P30 animals. RNA was extracted from the lateral and fourth ventricle choroid plexus, and cerebral cortex using the RNeasy Plus Micro kits (QIAGEN, Cat. No. 74034) following manufacturers' specifications. One μg of RNA was reverse transcribed using the iScript kit (Bio‐Rad, Cat. No. 1708891). The cDNA was amplified using primers indicated in Table 2 for genes of interest, *Sult1a1, Sult1c3, Sult4a1, Papss1* and *Papss2*, and reference gene *Sars1* (Eurofins Genomics), using the LC480 SYBR Green I Master (Roche Applied Science, 04707516001) in a LightCycler 480 (Roche Applied Science, RRID:SCR_018626). Melting‐curve analysis was performed to verify the amplification of a single product with a specific melting temperature. A negative PCR control without cDNA was included in all runs. Quantification cycle (Cq) values were calculated by the LightCycler 480 Software (Version 1.5.1) using the second derivative maximum analysis method. For each gene, a standard curve plotting the Cq values of at least five serial dilutions of a cDNA pool against the relative cDNA concentration was generated by non‐linear regression analysis and was used to determine the amplification efficiency. Sample‐to‐sample variations were corrected by normalising the data to the expression of *Sars1*, using the advanced mode of relative quantification analysis of the LightCycler 480 Software, with efficiency correction.

**TABLE 2:** Details of primers used for RT‐qPCR.

Gene target	Forward sequence	Reverse sequence
*Sult1a1*	ACCCTCTGCCTCAGCTACAT	ACAGCCCATCATGATCTCAA
*Sult1c3*	CAACGGGCCAACACCTATGA	TCCAGACCTGAGTTGAGGGG
*Sult4a1*	TATTCCTTGCTGGACACTCCG	CATCATCTCACTCCTCGGCTC
*Papss1*	ATTGTTGGACGAGACCCTGC	CATCGTCAGCACTTTGGCAC
*Papss2*	GGCCTCACCTCTGTGGAAAT	CAAACTCATCGTGCCTTGCC
*Sars1*	TGCCTTCCGTGAGTTGGTTT	ACAAACTCCACCTTGTCCATCA

### 2.4 Immunofluorescence Staining of Sulphotransferase in the Choroid Plexus

Lateral ventricle choroid plexuses were dissected, fixed in 4% paraformaldehyde and embedded in paraffin. 3 μm sections were collected. Slides were dewaxed and rehydrated through graded alcohols followed by phosphate buffered saline (PBS). Slides were then incubated with a protein blocker consisting of 10% normal goat serum +0.5% bovine serum albumin in PBS with 0.3% Triton for 2 h at room temperature. The primary antibody (rabbit polyclonal anti‐human SULT1A1, Abcam, Cat. No. ab155012) was diluted 1:100 (10 μg/mL) using 1% fish gelatine in PBS with 0.3% Triton and added to the slides for incubation overnight at 4°C. Following washes, the secondary antibody (goat anti‐rabbit Alexa Fluor 488, 2 μg/mL, Invitrogen, RRID:AB_143165) was applied for 2 h at room temperature. Finally, nuclei were counterstained with DAPI (4′,6‐diamidino‐2‐phenylindole) and slides were mounted in PermaFluor Aqueous Mounting Medium. Control sections were obtained by omitting the primary antibody and were included in every run. Images were captured using a Zeiss AxioImager M2 epifluorescence microscope at 3.2 s exposure for the Alexa Fluor 488 channel. All images were displayed using black and white values of 500 and 4000 respectively in ZEN (Zeiss, RRID:SCR_013672, Version 3.12) and no nonlinear adjustments were applied. Additional slides were stained with haematoxylin and phloxine for visualisation of gross tissue morphology.

### 2.5 Sulphotransferase Inhibition Assay

Sulphoconjugation of PNP to form PNPS by sulphotransferases in presence of an additional compound as potential inhibitor was investigated in P3 choroid plexus. PNP was chosen as it is a typical multi‐specific sulphotransferase substrate commonly used in inhibition studies (Nishimuta et al. 2007; Vietri et al. 2000), and it was reported to be recognised by a broad range of sulphotransferase isoforms (Gamage et al. 2006; Mizuma et al. 1983). Homogenates were prepared as described above. Multiple pools of homogenate were prepared from six animals each, and different inhibitors were tested in each pool. Reactions mixtures included 0.625 μM of PNP, 20 μM of PAPS and an inhibitor at a given concentration in 250 μL total volume (pH 7.6). A list of inhibitors and concentrations used are presented in Table 3. Control assays that contained only PNP were included in each run. Additionally, despite the very small volume of inhibitor solvents (methanol, ethanol and DMSO) used in assay mixtures, vehicle control experiments were first performed to ensure that any effects observed were due to the inhibitors themselves (Ma et al. 2003). Following incubation and precipitation of protein as described above, 400 μL supernatant was evaporated to dryness in a vacuum concentrator system, and subsequently reconstituted in acetonitrile for quantification of PNPS using HPLC. Activity was calculated as pmol of PNPS/min/mg of protein and results are presented as percentage (%) of activity compared to uninhibited controls.

**TABLE 3:** Compounds investigated in sulphotransferase inhibition experiments and concentrations used.

Compound		Concentration (μM)	Supplier and catalogue number
Quercetin	QCT	0.1	Sigma‐Aldrich, 337951
Mefenamic acid	MFA	0.1	Sigma‐Aldrich, 92574
Acetaminophen	AC	2500	Supelco, PHR1005
Bisphenol‐A	BPA	30	Sigma‐Aldrich, 239658
Dopamine	DA	50	Sigma‐Aldrich, H8502
B‐Estradiol	E2	1	Sigma‐Aldrich, E1024
3,5‐diiodo‐L‐Thyronine	T2	2.5	Sigma‐Aldrich, 719536

### 2.6 Live Isolated Choroid Plexus Sulphotransferase Activity

Lateral and fourth ventricle choroid plexuses were freshly isolated from P1 and P30 animals and warmed in HBSS with Mg/Ca at 37°C for 10 min. They were then transferred into 150 μL of warm HBSS (containing sulphate at 0.41 mM) supplemented with either 0.5 or 2.5 μM of PNP, or buffer alone. Following 1 h of incubation at 37°C under gentle agitation, choroid plexuses were rinsed in a large volume of 4°C fresh HBSS before being frozen for storage, and samples of incubation media were collected. To extract PNP and its metabolites from the choroid plexuses, tissue samples were homogenised in 100 μL of water and an aliquot sampled for protein measurement. The homogenate was then precipitated using an equal volume of acetonitrile as described above. After centrifugation, the supernatant was collected and analysed by LC–MS. Media solutions were analysed directly. Results are expressed as the amount of PNP or metabolites (pmol) per mg of protein in both choroid plexus and media.

### 2.7 Determination of Protein Concentration in Tissue Homogenate

Concentrations of protein in homogenate used in all assays described above were measured by an established method (Peterson 1977). Briefly, homogenates were diluted in the appropriate amount of water between 1:5 and 1:10 to maintain comparable concentrations. Reagents were added to diluted homogenates and bovine serum albumin standards and absorbance was read at 750 nm on a spectrophotometer. Protein concentrations were calculated from standard curves constructed between 0.05 and 0.5 mg/mL.

### 2.8 High‐Performance Liquid Chromatography (HPLC) and Liquid Chromatography–Mass Spectrometry (LC–MS) Analysis

Detection, identification and quantification of compounds generated in enzymatic reactions were performed using a Shimadzu HPLC system equipped with a dual‐pump liquid chromatograph (LC‐20ad), a system controller (CBM‐20A), and an autosampler (SIL‐40C). Chromatographic separation was achieved on a C_18_ column (4.6 × 250 mm, 5 μm; Interchim, US5C18HQ‐250/046) at room temperature. Chromatograms were extracted in LabSolutions (Shimadzu, Ver 5.109). Parameters specific to each type of assay are described below.

#### 2.8.1 Activity Assays

Concentrations of p‐Nitrophenol generated in sulphotransferase activity assays were determined using UV detection (Shimadzu, SPD‐10A UV–Vis Detector) at the optimised wavelength of 316 nm. The mobile phase consisted of aqueous 0.1% (*v*/*v*) trifluoroacetic acid (Sigma‐Aldrich, 302031) and acetonitrile (70:30, *v*/*v*) at the flow rate of 1 mL/min. The injection volume was 15 μL. Under these conditions, the retention time of 2‐naphthyl‐sulphate was 9.1 min and that of PNP was 12 min, and its quantification was based on a standard curve of PNP made of 6 levels of calibrator in the range of 1–100 μM.

#### 2.8.2 Inhibition Assays

For inhibition studies, PNPS from concentrated assay supernatant was measured using UV detection at the optimised wavelength of 290 nm. 40 μL of sample was eluted with a mobile phase consisting of aqueous 0.1% (*v*/*v*) trifluoroacetic acid and acetonitrile (70:30, *v*/*v*) at 1 mL/min. The retention time of PNPS was 8 min and its quantification was based on a standard curve in the range of 0.05–3 μM. Precaution was taken to ensure no interference was produced by inhibitor compounds or their solvents at the wavelength of interest that might obstruct quantification of PNPS.

#### 2.8.3 Live Choroid Plexus Assays

In tissue homogenate and media samples from the live choroid plexus assays, concentrations of PNPS were determined using a mass spectrometer (LCMS‐2050) with heated Dual Ion Source including electrospray ionisation and atmospheric pressure chemical ionisation (BIP facility). The reasons for switching to mass spectrometry instead of UV detection as described above for the inhibition assays were to improve sensitivity in quantitation and enable monitoring of additional metabolites which may be generated by live choroid plexuses. The injection volume was 20 μL and the mobile phase consisted of aqueous 1% (*v*/*v*) formic acid and acetonitrile (60:40, *v*/*v*) at the flow rate of 1 mL/min. Mass spectrometry experiments were conducted using a heated electrospray ionisation source in negative ion mode. The interface voltage was set to 2 kV and the desolvation temperature was 450°C. Acquisition of PNPS (218.00 m/z) and PNP (138.11 m/z) was performed using selected ion monitoring (SIM) mode at retention times of 13.8 and 6.7 min respectively (Figure S1). Following extraction of peak areas, sample concentrations were calculated using standards (2.4–1250 nM; PNP, 0.61–39.1 nM; PNPS).

### 2.9 Statistics and Data Analysis

Selection of animals and pooling of tissue were conducted arbitrarily. Both males and females were included in experiments. No a priori power analysis was performed to predetermine sample size as this is one of the first study to investigate sulphotransferase activity and expression in the developing rat choroid plexuses. Sample sizes were chosen with ethical and practical considerations, and were comparable to published enzymatic studies which employed similar methodologies (Kratzer et al. 2018; Strazielle and Ghersi‐Egea 1999). Statistical analyses and data presentation were performed using Prism (GraphPad Software Inc., RRID:SCR_002798, Version 11.0.0). Statistical differences across multiple age groups were determined using one‐way ANOVA (analysis of variance) with Tukey's multiple comparisons test. Effects of potential inhibitors were assessed with pair‐matched one‐way ANOVA for respective controls with Dunnett's multiple comparisons test. Comparisons between the lateral and fourth ventricle choroid plexuses were conducted using two‐tailed unpaired Student *t*‐tests with F tests. No tests for outliers were conducted. A *p*‐value of 0.05 or less was considered statistically significant. Results are presented as mean ± standard deviation. Details of normality tests are tabulated in Table S1, and details of all statistical methods and reports are tabulated in Table S2.

## 3 Results

### 3.1 Activity of Sulphotransferase in the Choroid Plexus Throughout Development

**FIGURE 2:**
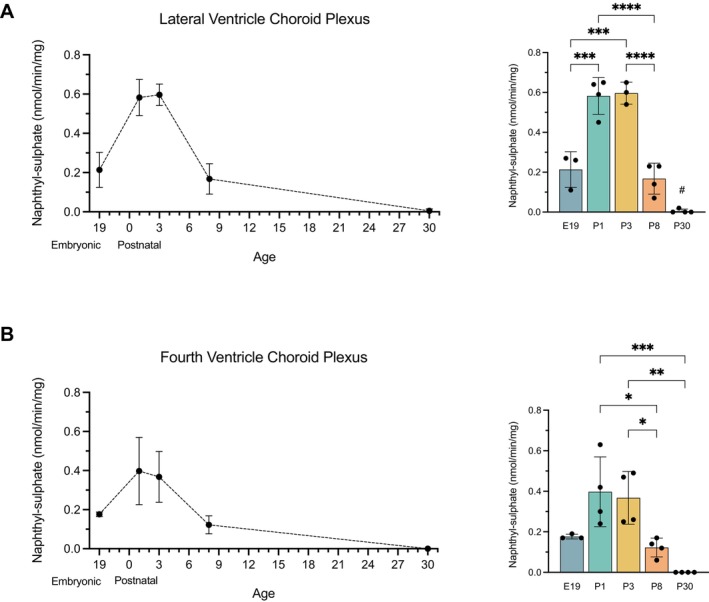
Sulphotransferase activity in the choroid plexuses throughout development. Developmental profile of specific activity towards 2‐naphthol (nmol/min/mg of protein) in the lateral ventricle (A) and fourth ventricle choroid plexus (B) from E19, P1, P3, P8 to P30 rats. Mean ± SD; *n* = 3–4. Individual values and statistically significant differences between ages are shown on the associated bar graphs. *p < 0.05, **p < 0.01, ***p < 0.001, ****p < 0.0001. # indicates significant difference with all other groups. 4VCP, fourth ventricle choroid plexus; LVCP, lateral ventricle choroid plexus.

To establish the profile of sulphotransferase specific activity in the lateral (LV) and fourth ventricle (4V) choroid plexus across development, sulphoconjugation of 2‐naphthol was measured in tissue homogenate from E19, P1, P3, P8 and P30 rats. A previously established and validated assay was utilised, whereby sulphotransferases catalysed sulphoconjugation reaction using PAPS as the sulphate donor, which was replenished by p‐nitrophenyl sulphate (Frame et al. 2000). To minimise the number of animals used, experiments were not designed to formally assess sex‐specific differences. However, we observed overlap in the choroid‐specific activities between tissues sampled from both sexes. Therefore, data from male and female were pooled. As illustrated in Figure 2A, the specific activity in the fetal LVCP was 0.21 ± 0.09 nmol/min/mg, which increased to the highest level in the newborns (0.58 ± 0.09; P1, 0.60 ± 0.06; P3). Postnatally, activity was found to decrease in an age‐dependent manner; it was lower in P8 and was virtually absent in P30. Likewise, a similar developmental pattern was observed in the 4VCP (Figure 2B). Comparison between the two plexuses showed that LVCP had significantly higher activity than 4VCP in P3 animals (*p* = 0.038).

**FIGURE 3:**
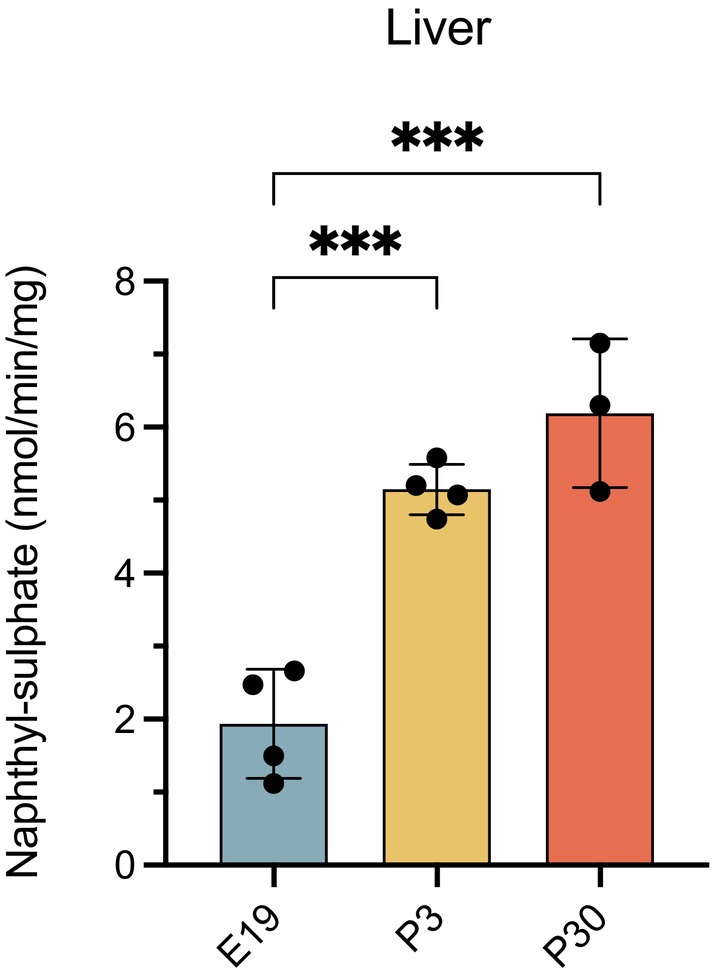
Sulphotransferase activity in the liver throughout development. Specific activity (nmol/min/mg of protein) in the liver in E19, P3 and P30 rats towards 2‐naphthol. Mean ± SD; *n* = 3–4. ***p < 0.001.

Sulphotransferase activity towards 2‐naphthol was also determined in the liver (Figure 3) as it is the major site of metabolism. Activities were markedly higher than those in age‐matched choroid plexuses as expected. The developmental profile was different from that seen in choroid plexuses, as the activities exhibited an age‐related increase from E19 (1.94 ± 0.75 nmol/min/mg) to P30 (6.19 ± 1.02 nmol/min/mg). Activities in the cerebral cortex at all ages and in the placenta of E19 fetuses were also investigated but were not detectable (data not shown, *n* = 2).

### 3.2 Developmental Expression of Genes Involved in Sulphoconjugation Mechanisms

The expression of *Sult1a1*, *Sult1c3* and *Sult4a1* was analysed by qRT‐PCR in the choroidal and cortical tissue at different ages. *Sult1a1* was selected as it is one of the most representative and widely expressed phenolic sulphotransferase (Hebbring et al. 2007), and *Sult4a1* is a highly expressed brain‐specific isoform (Falany et al. 2000). *Sult1c3* was investigated as it appeared to be more highly expressed than the rest of the isoforms in a rat choroid plexus transcriptomic dataset (Table S3, data from Qiu et al. (2025)). Some isoforms reported in the human choroid plexus, including SULT1A2 and SULT1A3, do not appear to have a rat ortholog (Sidharthan et al. 2013).

**FIGURE 4:**
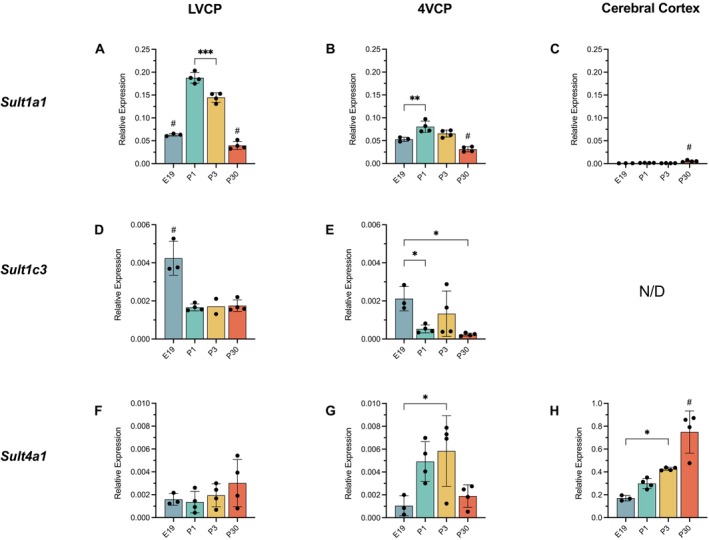
Expression of sulphotransferases in the choroid plexuses and cerebral cortex throughout development. mRNA expression of *Sult1a1* (A–C), *Sult1c3* (D, E) and *Sult4a1* (F–H) in the choroid plexus and cerebral cortex of E19, P1, P3 and P30 rats. Expression normalised against *Sars1* used as a reference gene. Mean ± SD; *n* = 3–4 except *Sult1c3*, P3 LVCP (*n* = 2). *p < 0.05, **p < 0.01, ***p < 0.001. # indicates significant difference with all other groups. 4VCP, fourth ventricle choroid plexus; LVCP, lateral ventricle choroid plexus; N/D, not detected. Note different *y*‐axis scales between (F), (G) and (H), and that reported relative expression levels in *y*‐axis scale cannot be compared between genes.

The most predominant choroidal sulphotransferase was *Sult1a1*, with a quantification cycle (Cq) of 23.36 ± 0.17 at P1, compared to *Sult1c3* and *Sult4a1* which had a Cq of 30.96 ± 0.18 and 33.03 ± 0.71, respectively. *Sult1a1* exhibited higher expression in the two neonatal age groups compared to fetal and P30 rats (Figure 4A,4B). Additionally, it was more expressed at P1 than at P3 in the LVCP (0.19 ± 0.01; P1, 0.14 ± 0.01; P3), but not the 4VCP. Comparison between the two plexuses revealed significantly higher *Sult1a1* expression in the LVCP than 4VCP at all age groups other than P30 (*p* = 0.032; E19, *p* < 0.0001; P1, *p* < 0.0001; P3, *p* = 0.15, P30). Another isoform, *Sult1c3*, showed a different developmental pattern as its expression was highest in the E19 LVCP, and remained consistent in all postnatal age groups (0.0042 ± 0.0009; E19, 0.0015–0.0022; P1–P30, Figure 4D). Its expression in the 4VCP appeared to follow the same pattern (Figure 4E). Expression of *Sult4a1* was not statistically different between any age groups investigated in the lateral ventricle choroid plexus (Figure 4F) and showed a peak in expression at P3 in the fourth ventricle choroid plexus (Figure 4G). In cerebral cortex (Figure 4H), its expression was prominent and increased with age, with the highest level found in P30 (0.75 ± 0.19), showing a Cq of 23.18 ± 0.64. In comparison, *Sult1a1* had a Cq point of 27.74 ± 0.59 (Figure 4C) and *Sult1c3* was not detected at all in the brain.

**FIGURE 5:**
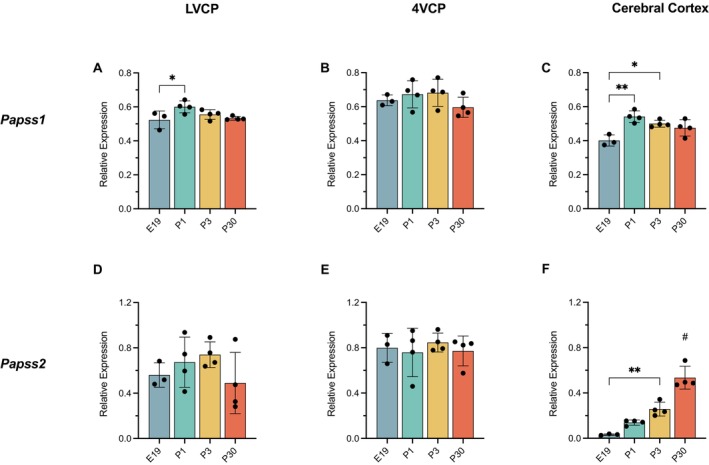
Expression of PAPS synthases in the choroid plexuses and cerebral cortex throughout development. mRNA expression of *Papss1* (A–C) and *Papss2* (D–F) in the choroid plexus and cerebral cortex of E19, P1, P3 and P30 rats. Expression normalised against *Sars1* used as a reference gene. Mean ± SD; *n* = 3–4. *p < 0.05, **p < 0.01. # indicates significant difference with all other groups. 4VCP, fourth ventricle choroid plexus; LVCP, lateral ventricle choroid plexus. Note that reported relative expression levels reported in *y*‐axis scales cannot be compared between genes.

Developmental expression of 3′‐phosphoadenosine 5′‐phosphosulphate synthetases 1 & 2 (*Papss1 & 2*), enzymes which synthesise the universal sulphate donor PAPS for all sulphotransferases, was also examined (Figure 5). Choroidal expression of both isoforms displayed developmental patterns different from those observed for the main *Sult1a1* isoform as they were constant between age groups apart from a small but statistically significant increase in the LVCP expression of *Papss1* between E19 and P1 (0.52 ± 0.05; E19, 0.60 ± 0.04; P1, Figure 5A). In the cerebral cortex, expression of *Papss1* also increased slightly from 0.40 ± 0.03 in fetuses to 0.54 ± 0.03 at P1 and 0.50 ± 0.02 at P3 (Figure 5C). The cortical expression of *Papss2* increased with age and was highest in P30 animals as observed for the *Sult4a1* cortical isoform (Figure 5F).

### 3.3 Cellular Localisation of Choroidal Sulphotransferase

**FIGURE 6:**
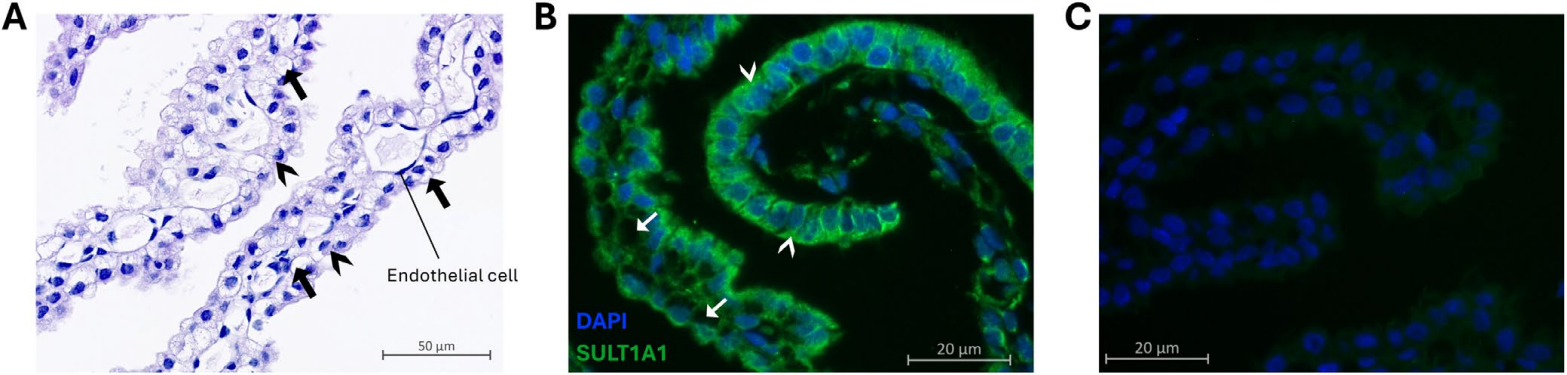
Immunostaining of sulphotransferase in lateral ventricle choroid plexus at P3. (A) Haematoxylin and phloxine staining of section. Note extensive glycogen deposits (arrows) typically found in choroid plexus epithelial cells during development. Arrowheads indicate cytoplasmic space. A representative endothelial cell of the choroidal capillary is shown. (B) Immunostaining of SULT1A1 (green) with counterstain of nuclei (blue). Locations of representative glycogen stores and cytoplasm are indicated. (C) Control section obtained by omitting the primary antibody showing no detectable signal. Scale bar is 50 μm in (A) and 20 μm in (B) and (C).

Immunofluorescence staining was performed using an antibody directed against SULT1A1 which may also cross‐react with other SULT isoenzymes. In P3 animals, the signal was mainly restricted to the epithelial cells of the choroid plexuses (Figure 6). It was observed in the cytoplasm of the cells, as expected, and did not overlap with intracellular glycogen stores, which are typically present during development (Figure 6A,B). Similar but weaker staining was observed in choroid plexuses of E19 fetuses, and no staining above background was observed in choroid plexuses of P30 animals (Data not shown).

### 3.4 Identification of Choroidal Sulphotransferase Inhibitors

The sulphation of p‐nitrophenol, a multi‐specific substrate for sulphotransferases at a concentration of 0.63 μM, chosen to reflect its lower Km (Hirshey and Falany 1990; Nakamura et al. 1987; Zhu et al. 1996), was measured in the presence of other compounds as a mean to identify possible substrates or inhibitors of choroidal sulphotransferases. Substances tested represent a diverse range of chemically distinct compounds, displaying different biological functions (Table S4). They include nonsteroidal anti‐inflammatory drug mefenamic acid and antipyretic and analgesic drug acetaminophen/paracetamol; endogenous hormones dopamine, b‐estradiol and T2; quercetin, a natural flavonoid; and bisphenol‐A, an environmental toxin. Quercetin and mefenamic acid are known sulphotransferase inhibitors while the other compounds are potential substrates (Cook et al. 2019; Mesia‐Vela and Kauffman 2003). The concentrations used are tabulated in Table 2.

**FIGURE 7:**
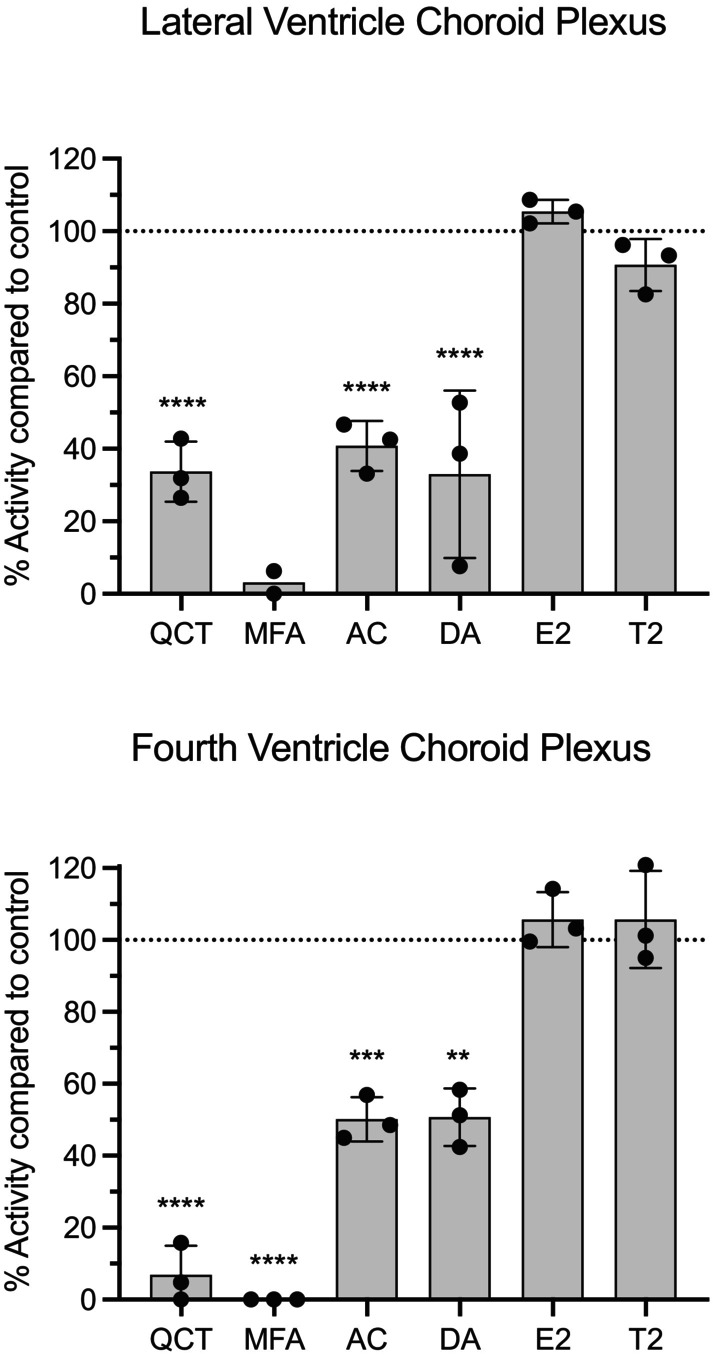
Inhibitory capacity of selected compounds towards p‐nitrophenol in the choroid plexuses. Percentage of activity in presence of a potential sulphotransferase inhibitor compared to control in the lateral ventricle (A) and fourth ventricle (B) choroid plexuses of P3 rats. Concentrations used for each test compound are provided in Table 2. Mean ± SD, *n* = 3 except lateral ventricle choroid plexus, MFA, where *n* = 2 (excluded from statistical tests). **p < 0.01, ***p < 0.001, ****p < 0.0001. AC, acetaminophen; DA, dopamine; E2, B‐estradiol; MFA, mefenamic acid; QCT, quercetin; T2, 3,5‐diiodo‐L‐thyronine.

Choroid plexuses (LV & 4V) homogenates from P3 pups were used as it was the height of sulphotransferase activity in the choroid plexus (Figure 2). Results are represented as % of activity in presence of the potential inhibitor compared to uninhibited control in Figure 7 Presence of quercetin significantly reduced sulphotransferase activity to 33.7% ± 8.3% of that in the control in the LVCP (Figure 7A), and to a greater extent in the 4VCP (6.9% ± 8.1%, Figure 7B). For the therapeutic compounds tested, mefenamic acid completely inhibited activity in the 4VCP and almost completely in the LVCP (0.0% & 6.3%). Acetaminophen resulted in a moderate reduction of sulphoconjugation of PNP to 40%–50% in both tissues. Out of the three endogenous substances investigated, only dopamine showed statistically significant reduction in sulphotransferase activity in the choroid plexuses (33.0% ± 23.1%; LVCP, 50.7% ± 8.0%; 4VCP, Figure 7A,B).

**FIGURE 8:**
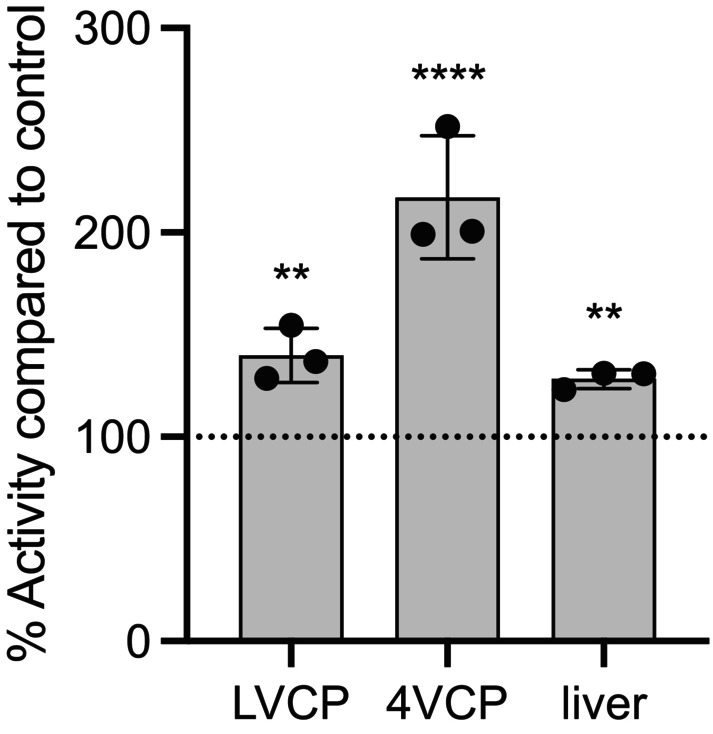
Effect of bisphenol‐A on sulphotransferase activity in the choroid plexuses and liver. Data are expressed as percentage of activity towards p‐nitrophenol in presence of bisphenol‐A compared to control in the lateral ventricle and fourth ventricle choroid plexus and liver of P3 rats. Mean ± SD; *n* = 3. **p < 0.01, ****p < 0.0001. 4VCP, fourth ventricle choroid plexus; LVCP, lateral ventricle choroid plexus.

Interestingly, it was found that co‐incubation with bisphenol‐A appeared to increase the extent of sulphoconjugation of PNP in both the choroid plexuses and liver. As illustrated in Figure 8, activity increased to 140.0% ± 13.2%, 217.2% ± 30.1% and 128.3% ± 4.6% in LVCP, 4VCP and liver respectively (*p* = 0.0026, *p* < 0.0001, *p* = 0.0011). Potential explanations and implications for this observation are considered in the Discussion.

### 3.5 Sulphoconjugate Production by Live Isolated Choroid Plexuses

To examine the choroidal activity of sulphotransferase in a more physiological condition which closer resembles the in vivo environment, the capacity of intact choroid plexuses freshly isolated from P1 and P30 animals to metabolise PNP was assessed. Live choroid plexuses were exposed to PNP (0.5 or 2.5 μM), and PNPS quantified in both the tissue and medium by LC–MS.

**FIGURE 9:**
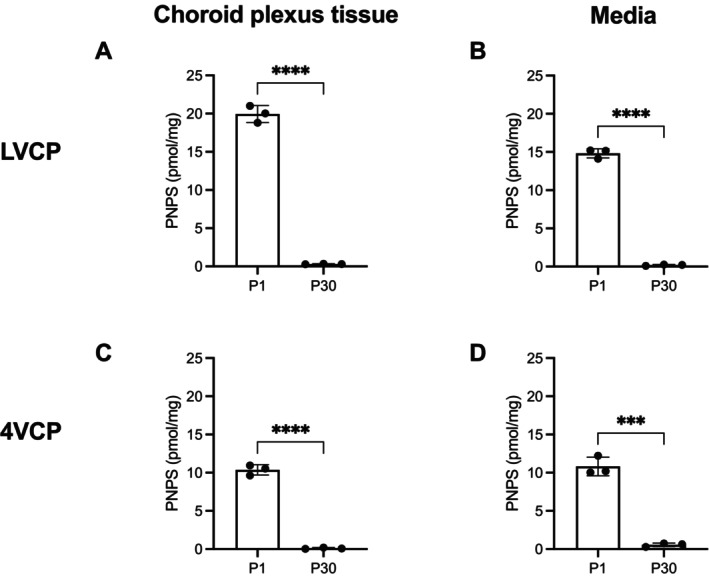
Sulphoconjugation of PNP in live isolated choroid plexuses at P1 & P30. Production of PNPS by freshly isolated lateral ventricle choroid plexus (A, B) and fourth ventricle choroid plexus (C, D) at P1 and P30. Incubation media contained PNP at 2.5 μM. At the end of one‐hour incubation, amount of PNPS measured in the choroid plexus tissues (A, C) and media (B, D) were normalised to mg of protein in the respective choroid plexus. Mean ± SD; *n* = 3–4. ***p < 0.001, ****p < 0.0001. 4VCP, fourth ventricle choroid plexus; LVCP, lateral ventricle choroid plexus.

Following 1 h of incubation with 2.5 μM of PNP, PNPS was found in the tissues themselves at 20.0 ± 1.1 pmol/mg of protein at P1, compared to 0.23 ± 0.16 pmol/mg of protein at P30 in the LVCP (Figure 9A). In the media, amounts present normalised to choroid plexus protein concentration were 17.0 ± 4.0 pmol/mg of protein for P1 and 0.2 ± 0.07 pmol/mg of protein for P30 (*p* < 0.0001, Figure 9B). Likewise, PNPS concentrations in the 4VCP tissue were 10.4 ± 0.7 and 0.08 ± 0.09 pmol/mg of protein (P1 vs. P30), and 9.2 ± 1 and 0.41 ± 0.34 pmol/mg of protein (P1 vs. P30) in the 4VCP media (Figure 9C,D). The substantially greater extent of sulphoconjugation in the choroid plexus of newborn rats than older animals was therefore in agreement with prior findings from the homogenate enzymatic assays (Figure 2).

**FIGURE 10:**
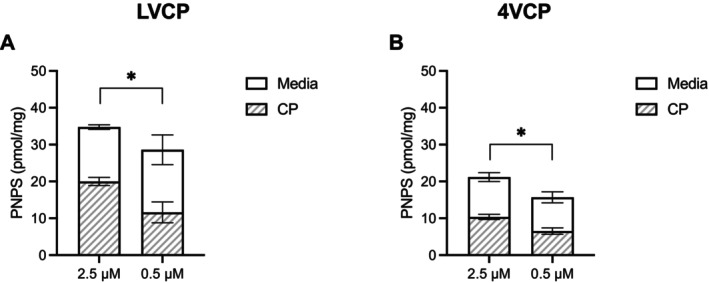
Effect of PNP concentration on its sulphoconjugation in live isolated choroid plexuses at P1. Production of PNPS by freshly isolated lateral ventricle choroid plexus (A) and fourth ventricle choroid plexus (B) at P1. Incubation media contained PNP at 2.5 or 0.5 μM. At the end of one‐hour incubation, the amount of PNPS measured in the choroid plexus tissues (hatched bar) and media (plain bar) was normalised to mg of protein in the respective choroid plexus. Mean ± SD; *n* = 3. *p < 0.05. 4VCP, fourth ventricle choroid plexus; LVCP, lateral ventricle choroid plexus.

The substrate concentration‐dependency of sulphoconjugation was further tested by exposing choroid plexuses from P1 pups to PNP at 5 times lower concentration. Combining the tissue and medium samples, comparison between the two concentrations revealed that amount of PNPS produced by the LVCP were 34.8 ± 1.7 pmol/mg from 2.5 μM of PNP, which was only 1.2‐fold higher than the amount generated from 0.5 μM of PNP (28.6 ± 1.8 pmol/mg, Figure 10A). In the 4VCP, increasing the PNP concentration from 0.5 to 2.5 μM resulted in 1.4 times more total PNPS (15.7 ± 2.3 pmol/mg; 0.5 μM, 21.2 ± 1.8 pmol/mg; 2.5 μM, Figure 10B).

## 4 Discussion

The present work utilised a rat model to investigate developmental changes in brain sulphotransferases, which are involved in detoxification of xenobiotics and inactivation of phenolic endogenous molecules (M. W. Coughtrie 2016). The first part of the study focused on characterising the developmental profiles of activity of sulphotransferases along with their expression in the lateral and fourth ventricle choroid plexuses, with comparisons made with the cerebral cortex and liver. The developmental stages investigated included late fetal age E19 to young adolescence P30.

Results illustrated in Figure 2 showed that sulphotransferase activity increased between the late fetal stage and newborns, reaching a peak at P1 and P3 in both fourth and lateral ventricle choroid plexuses, and subsequently decreased towards adulthood. The marked contrast in levels of activity between newborns and young adults was reinforced in a separate assay system using live choroid plexuses and p‐nitrophenol as substrate (Figures 9 and 10). Out of the three sulphotransferase isoforms investigated by RT‐qPCR, the developmental expression pattern of *Sult1a1* (Figure 4A,B) resembled that of enzymatic activity, signifying a positive correlation between mRNA expression and functional outcomes. The close agreement is likely due to the relatively high expression of *Sult1a1* compared to the other isoforms in the choroid plexus, and the specificity of the assay employed, which was developed to examine predominantly SULT1A activity by using 2‐naphthol as the test substrate (Frame et al. 2000; Ozawa et al. 1994). The developmental pattern with a neonatal spike in activity and expression appeared to differ from many other phase II detoxifying enzymes expressed in the rat choroid plexus, as most isoforms of UDP‐glucuronosyltransferase and glutathione S‐transferase have been reported to show either up‐ or down‐regulation with age from E19 to adulthood (Kratzer et al. 2013). This distinctive profile may indicate particular significance of SULT1A1 in that specific early postnatal window of development. This timing coincides with increased concentration of sulphate in circulation during development; maternal sulphate levels increased throughout gestation despite the effect of haemodilution lowering concentrations of most plasma ions. Moreover, sulphate is actively transported across the placenta into the fetuses (Cole et al. 1984, 1985). After birth, concentration of serum sulphate appeared to be higher in infants than in three‐years‐old children, and similarly higher in kittens between 1 and 7 days compared to adult cats (Cole et al. 1982; Van Harreveld et al. 1966). These observations highlight the developmental significance of sulphation in general and support the notion that increased choroidal sulphotransferase activity during early postnatal stages could be physiologically sustained.

Using freshly isolated P1 live choroid plexus in an assay system where PAPS regeneration is self‐sustained only from sulphate present in the medium (Figures 9 and 10), a more in vivo situation was represented as sulphoconjugation would not only depend on the activity of sulphotransferases but also on the availability of the sulphate donor PAPS (Burkart et al. 2000; Klaassen and Boles 1997; Lee et al. 2006). Present results demonstrated that the neonatal choroid plexuses were capable of synthesising PAPS from PAP and inorganic sulphate under the assay timeframe, showing that elevated activity of sulphotransferase in choroid plexuses during this window of development could be physiologically supported. Detection of metabolite in the surrounding media, in addition to the tissues themselves (Figure 9), indicates that the sulphoconjugate is likely extruded from the cells, suggesting the involvement of potential efflux mechanisms. Two concentrations of PNP (0.5 and 2.5 μM) were tested (Figure 10). Results demonstrated that the decreasing concentration did not lead to a proportional decrease in PNPS production, as the total PNPS generated was only 1.2‐fold lower at 0.5 μM, indicating that at media concentration as low as 0.5 μM, the intracellular concentration of PNP was already approaching the maximal velocity of the choroidal sulphation machinery. This is consistent with the reported Km values in the low μM range for PNP at concentrations below those that induce substrate inhibition (Hirshey and Falany 1990; Nakamura et al. 1987; Zhu et al. 1996). Additionally, sulphoconjugation likely faces competition with alternative metabolic pathways in this assay. Combining amounts of PNPS generated in choroid plexus tissue and in the medium, it was found that only 7% and 3% of the amount of PNP present at the start of incubation has been recovered as PNPS, for the 0.5 and 2.5 μM concentrations respectively. Preliminary analyses of these samples revealed that a substantial amount of PNP‐glucuronide was potentially synthesised, consistent with an in vivo study reporting that PNP is more prone to conjugation with glucuronic acid than with sulphate in rats, likely through liver metabolism (Higaki et al. 2003). Such difference confirms that choroidal sulphotransferase is likely a high‐affinity and low‐capacity system, and may suggest greater efficacy of the glucuronide conjugation pathway at metabolising exogenous phenolic compounds in the choroid plexus. However, there may be substrates more specific or exclusive to sulphate conjugation such as minoxidil, and some catecholamines and peptide hormones.

To better understand the potential significance of the neonatal peak in sulphotransferase activity within the choroid plexus, inhibition studies were conducted as an attempt to identify their possible substrates or inhibitors (Table S4). Activity assays were performed by co‐incubation of p‐nitrophenol with another compound to explore their interactions (Figures 7 and 8). P‐nitrophenol is a multi‐specific substrate which has been used in other inhibition studies (Nishimuta et al. 2007; Vietri et al. 2000). Although it is a typical substrate for SULT1A1, its catalysis has been demonstrated with other isoforms, albeit with relatively lower affinity (Chapman et al. 2004; Duffel et al. 2001). Inhibition of PNPS formation could indicate substrate competition, non‐competitive inhibition of sulphotransferases, or competition for PAPS by two sulphotransferase isoforms (Klaassen and Boles 1997; Mulder 1981).

Quercetin is one of the most abundant flavonoids found in many fruits and vegetables, and beverages such as red wine. It is well known for its potent inhibition of sulphotransferase (James and Ambadapadi 2013; Mesia‐Vela and Kauffman 2003; Nishimuta et al. 2007), reducing the sulphation of prototypical substrates across several different isoforms such as PNP, dopamine, acetaminophen, minoxidil, E2 and dehydroepiandrosterone with IC_50_ in the μM‐nM range (Wang and James 2006). In the P3 choroid plexus, quercetin also showed 70%–90% inhibition of PNP sulphation at 0.1 μM, as illustrated in Figure 7. The effects of nonsteroidal anti‐inflammatory drug mefenamic acid and antipyretic and analgesic drug acetaminophen were examined. At 0.1 μM, mefenamic acid resulted in almost complete inhibition of sulphotransferase activity in the choroid plexus, consistent with findings reported in fetal and adult human liver (Vietri et al. 2000, 2001). Mefenamic acid is not typically considered as a sulphotransferase substrate but a potent inhibitor specific to SULT1A1 (Cook et al. 2019). In contrast, presence of acetaminophen only led to moderate inhibition even at a high concentration of 2.5 mM. It likely inhibited PNPS formation by competition as it is a substrate recognised by several sulphotransferase isoforms, suggesting the capacity of developing choroidal tissue to metabolise drugs such as acetaminophen (Adjei et al. 2008; Yamamoto et al. 2015).

Endogenous sulphotransferase substrates, dopamine, E2 and T2, were evaluated for their interaction with choroidal sulphotransferases. These have been shown to be predominantly catalysed by SULT1A3, SULT1E1 and SULT1B1/1C1, respectively, though overlapping specificity with other isoforms has been reported (Chapman et al. 2004; Gamage et al. 2006; Visser et al. 1998). Dopamine induced significant inhibition in the present study (Figure 7), but no specific activity towards dopamine was detected in the human fetal choroid plexus (Richard et al. 2001). This may be due to species‐dependent differences in the expression and distribution of the relevant sulphotransferases as dopamine sulphation exhibited differential regional variation between human and rat brain (Richard et al. 2001; Rivett et al. 1984). Dopamine‐sulphate has been demonstrated to exhibit much reduced binding to the D2 receptors (Werle et al. 1988), thus sulphation may be a mechanism to deactivate excess dopamine and regulate its rapidly fluctuating levels in the central nervous system. Since disruption to developmentally regulated dopaminergic signalling has been associated with altered neural circuit formation and neurodevelopmental disorders such as autism spectrum disorder (Lu et al. 2024), the potential role of dopamine metabolism at choroid plexuses warrants further investigation. Finally, no inhibitory effects were observed in presence of E2 & T2, in contrast to the human choroid plexus which showed some activity towards T2 (Richard et al. 2001; Stanley et al. 2005). This may point to the lack of relevant isoforms for these hormones in the developing rat choroid plexus, for example, Sult1e1, Sult1b1 and Sult1c1, in line with their lack of expression in a rat transcriptomic choroidal database (Qiu et al. 2025). Additionally, they may exhibit relatively weaker activity towards PNP, as has been demonstrated for human sulphotransferases (Chapman et al. 2004).

Bisphenol‐A is a known endocrine disrupter widely used in industry and can be found in polycarbonate and epoxy resins products (Rochester 2013). Moreover, it has been shown to enter the brain and is associated with neurodevelopmental deficits (Denuzière and Ghersi‐Egea 2022). Sulphoconjugation of this molecule has been demonstrated using recombinant human phenol sulphotransferase (Suiko et al. 2000), and it can be inhibited by PNP, dehydroepiandrosterone (a SULT2A1 substrate) and quercetin, but not by dopamine and oestrogen in human liver cytosol (Shimizu et al. 2002), indicating that bisphenol‐A could be recognised by several sulphotransferase isoforms. Interestingly, present findings revealed that not only was PNP sulphation not inhibited by bisphenol‐A, but it appeared to be enhanced in both choroid plexus and liver tissues (Figure 8). This particular interaction between bisphenol‐A and PNP in choroidal sulphotransferase has not been reported before; but one study described a similar increase in dehydroepiandrosterone sulphoconjugation in presence of bisphenol‐A (Shimizu et al. 2002). A possible speculation for this apparent stimulatory phenomenon may be suppression of substrate inhibition effect. Substrate inhibition is known to occur in many sulphotransferases whereby at high substrate concentration, a second non‐catalytic binding site can be occupied by another substrate molecule, obstructing the catalytic site or affecting its efficacy (Gamage et al. 2006; James 2014; Wang and James 2006). A study suggested that manganese markedly enhanced sulphation of L‐dopa by displacing the binding of a second L‐dopa molecule, thereby abolishing effects of substrate inhibition (Barnett et al. 2004).

Compared to the choroid plexuses, specific activity in the liver was much higher at all ages examined. Even at its peak level in the P3 choroid plexus, activity was still approximately 7 times higher in the liver tissue (Figures 2 and 3). Likewise, sulphotransferase activity in the fetal human liver at 16 weeks of gestation was shown to be about 8 times greater than that in the 15–20 weeks choroid plexus (Richard et al. 2001). In contrast, the specific activity of glutathione S‐transferase was twice greater in the P2 rat choroid plexus than the age‐matched liver (Kratzer et al. 2018). This may imply that unlike the choroidal glutathione conjugation pathway, which serves as an enzymatic barrier for the developing brain during a period when hepatic xenobiotic metabolism is not fully mature, the sulphoconjugation system may have certain specialised functions local to the blood‐CSF barrier while the liver provides primary metabolic capacity. Liver sulphotransferase activity also exhibited developmental increase (Figure 3), which is in accordance with another study showing that activity towards p‐nitrophenol in the rat liver was low during gestation but increased rapidly in the perinatal period (Carroll 1969). Finally, another tissue type tested was the late gestation (E19) placenta which exhibited no detectable sulphotransferase activity. Likewise in humans, placental activity has also been reported to be limited, being approximately ten times less than the fetal liver (Pacifici et al. 1988).

In contrast to the choroid plexuses, no detectable sulphotransferase activity towards 2‐naphthol was found in the cerebral cortex at any age groups in the current study, in spite of abundant expression of *Sult4a1* in the cerebral cortex at P30 (Figure 4H). *Sult4a1* was first identified by Falany et al. (2000), who described a novel and highly expressed brain‐specific sulphotransferase in humans and rats. Its expression was detected in the rat brain one day after birth and increased until 7 days old when it reached adult levels (Falany et al. 2000), which is in good agreement with present findings. Later study further characterised this sulphotransferase and demonstrated its immunoreactivity throughout the rat brain, particularly in regions such as the prefrontal cortex, subthalamic nucleus and brainstem (Liyou et al. 2003). However, no substrate has been defined for SULT4A1 to date and its function remains uncertain (Allali‐Hassani et al. 2007; Minchin et al. 2008). The much more reduced activity of sulphotransferases in the cerebral cortex is likely a reflection of low cortical *Sult1a1* expression, reinforcing the choroidal specificity of its functions in the developing central nervous system.

## 5 Conclusion and Implications

In summary, the present study established the distinct developmental profiles of sulphotransferase activity and expression in the choroid plexuses of developing rats, characterised by a marked increase during the neonatal period. The age‐ and tissue‐specific expression of sulphotransferases in the choroid plexus, together with their interaction with xenobiotics and neuroendocrine factors, allude to a diverse range of functions for these enzymes at the blood–CSF barrier during a critical developmental window, which may include protection against potentially harmful substances and regulation of neurotransmitters. The observed modulation of sulphotransferase activity by commonly used drugs, natural dietary compounds, or environmental toxins implies that their exposure during development may perturb sulphotransferase‐mediated choroidal biological processes, potentially altering the tightly regulated internal environment of the brain which is crucial for appropriate neurodevelopment.

## Author Contributions

**Jean‐François Ghersi‐Egea:** conceptualization, methodology, investigation, formal analysis, supervision, funding acquisition, writing – review and editing. **Nathalie Strazielle:** conceptualization, investigation, formal analysis, supervision, writing – review and editing. **Fiona Qiu:** conceptualization, methodology, investigation, formal analysis, writing – original draft, writing – review and editing. **Anne Denuziere:** conceptualization, methodology, supervision, writing – review and editing.

## Funding

This work was supported by ERA‐Net NEURON ChorNEXUS, ANR‐24‐NEU2‐0002‐04.

## Ethics Statement

Animal care and procedures were conducted according to the guidelines approved by the French Ethical Committee (decree 87–848) and by the European Community directive 86 to 609‐EEC.

## Conflicts of Interest

The authors declare no conflicts of interest.

## Supporting information


**Table S1:** Assessments of normality of data distribution using the Shapiro–Wilk test, including the Shapiro–Wilk test statistic (W) and corresponding *p*‐value for each group in either ANOVA or *t*‐tests.
**Table S2:** Summary of statistical analyses including test statistics, degrees of freedom, *p* values and post hoc comparisons for all figures.
**Table S3:** Expression of sulphotransferases (Counts per million, CPM) in adult rat choroid plexus and brain cortex by RNA‐seq.
**Table S4:** Details of compounds investigated as potential substrate or inhibitors in sulphotransferase inhibition experiments, including their sources and functions, as well as chemical structures.
**Figure S1:** Extracted ion chromatograms of PNP (138.11 m/z, upper) and PNPS (218.00 m/z, lower) in a sample from live choroid plexus sulphotransferase activity assay.

## Data Availability

The data that support the findings of this study are available from the corresponding author upon reasonable request.

## References

[jnc70533-bib-0001] Adjei, A. A. , A. Gaedigk , S. D. Simon , R. M. Weinshilboum , and J. S. Leeder . 2008. “Interindividual Variability in Acetaminophen Sulfation by Human Fetal Liver: Implications for Pharmacogenetic Investigations of Drug‐Induced Birth Defects.” Birth Defects Research Part A: Clinical and Molecular Teratology 82, no. 3: 155–165. 18232020 10.1002/bdra.20535

[jnc70533-bib-0002] Allali‐Hassani, A. , P. W. Pan , L. Dombrovski , et al. 2007. “Structural and Chemical Profiling of the Human Cytosolic Sulfotransferases.” PLoS Biology 5, no. 5: e97. 17425406 10.1371/journal.pbio.0050097 PMC1847840

[jnc70533-bib-0003] Alnouti, Y. , and C. D. Klaassen . 2006. “Tissue Distribution and Ontogeny of Sulfotransferase Enzymes in Mice.” Toxicological Sciences 93, no. 2: 242–255. 16807285 10.1093/toxsci/kfl050

[jnc70533-bib-0004] Barnett, A. C. , S. Tsvetanov , N. Gamage , J. L. Martin , R. G. Duggleby , and M. E. McManus . 2004. “Active Site Mutations and Substrate Inhibition in Human Sulfotransferase 1A1 and 1A3.” Journal of Biological Chemistry 279, no. 18: 18799–18805. 14871892 10.1074/jbc.M312253200

[jnc70533-bib-0005] Bauer, H.‐C. , H. Bauer , A. Lametschwandtner , A. Amberger , P. Ruiz , and M. Steiner . 1993. “Neovascularization and the Appearance of Morphological Characteristics of the Blood‐Brain Barrier in the Embryonic Mouse Central Nervous System.” Developmental Brain Research 75, no. 2: 269–278. 8261616 10.1016/0165-3806(93)90031-5

[jnc70533-bib-0006] Burkart, M. D. , M. Izumi , E. Chapman , C.‐H. Lin , and C.‐H. Wong . 2000. “Regeneration of PAPS for the Enzymatic Synthesis of Sulfated Oligosaccharides.” Journal of Organic Chemistry 65, no. 18: 5565–5574. 10970295 10.1021/jo000266o

[jnc70533-bib-0007] Carroll, J. 1969. “Phenolsulfotransferase in the Developing Rat.” American Journal of Clinical Nutrition 22, no. 8: 978–985. 5805227 10.1093/ajcn/22.8.978

[jnc70533-bib-0008] Chapman, E. , M. D. Best , S. R. Hanson , and C. H. Wong . 2004. “Sulfotransferases: Structure, Mechanism, Biological Activity, Inhibition, and Synthetic Utility.” Angewandte Chemie International Edition 43, no. 27: 3526–3548. 15293241 10.1002/anie.200300631

[jnc70533-bib-0009] Cole, D. , L. Baldwin , and L. Stirk . 1985. “Increased Serum Sulfate in Pregnancy: Relationship to Gestational Age.” Clinical Chemistry 31, no. 6: 866–867. 3995766

[jnc70533-bib-0010] Cole, D. E. , L. S. Baldwin , and L. J. Stirk . 1984. “Increased Inorganic Sulfate in Mother and Fetus at Parturition: Evidence for a Fetal‐To‐Maternal Gradient.” American Journal of Obstetrics and Gynecology 148, no. 5: 596–599. 6702922 10.1016/0002-9378(84)90755-5

[jnc70533-bib-0011] Cole, D. E. , J. Shafai , and C. R. Scriver . 1982. “Inorganic Sulfate in Cerebrospinal Fluid From Infants and Children.” Clinica Chimica Acta 120, no. 1: 153–159. 10.1016/0009-8981(82)90086-9 7067135

[jnc70533-bib-0012] Cook, I. , T. Wang , and T. S. Leyh . 2019. “Isoform‐Specific Therapeutic Control of Sulfonation in Humans.” Biochemical Pharmacology 159: 25–31. 30423313 10.1016/j.bcp.2018.11.010 PMC6625639

[jnc70533-bib-0013] Coughtrie, M. 2002. “Sulfation Through the Looking Glass—Recent Advances in Sulfotransferase Research for the Curious.” Pharmacogenomics Journal 2, no. 5: 297–308. 12439736 10.1038/sj.tpj.6500117

[jnc70533-bib-0014] Coughtrie, M. W. 2016. “Function and Organization of the Human Cytosolic Sulfotransferase (SULT) Family.” Chemico‐Biological Interactions 259: 2–7. 27174136 10.1016/j.cbi.2016.05.005

[jnc70533-bib-0015] Denuzière, A. , and J.‐F. Ghersi‐Egea . 2022. “Cerebral Concentration and Toxicity of Endocrine Disrupting Chemicals: The Implication of Blood‐Brain Interfaces.” Neurotoxicology 91: 100–118. 35436567 10.1016/j.neuro.2022.04.004

[jnc70533-bib-0016] Duffel, M. W. , A. D. Marshall , P. McPhie , V. Sharma , and W. B. Jakoby . 2001. “Enzymatic Aspects of the Phenol (Aryl) Sulfotransferases.” Drug Metabolism Reviews 33, no. 3–4: 369–395. 11768773 10.1081/dmr-120001394

[jnc70533-bib-0017] Ek, C. J. , K. M. Dziegielewska , H. Stolp , and N. R. Saunders . 2006. “Functional Effectiveness of the Blood‐Brain Barrier to Small Water‐Soluble Molecules in Developing and Adult Opossum ( *Monodelphis domestica* ).” Journal of Comparative Neurology 496, no. 1: 13–26. 16528724 10.1002/cne.20885 PMC2634607

[jnc70533-bib-0018] Falany, C. N. 1991. “Molecular Enzymology of Human Liver Cytosolic Sulfotransferases.” Trends in Pharmacological Sciences 12: 255–259. 1949190 10.1016/0165-6147(91)90566-b

[jnc70533-bib-0019] Falany, C. N. 1997. “Enzymology of Human Cytosolic Sulfotransferases.” FASEB Journal 11, no. 4: 206–216. 9068609 10.1096/fasebj.11.4.9068609

[jnc70533-bib-0020] Falany, C. N. , X. Xie , J. Wang , J. Ferrer , and J. L. Falany . 2000. “Molecular Cloning and Expression of Novel Sulphotransferase‐Like cDNAs From Human and Rat Brain.” Biochemical Journal 346, no. 3: 857–864. 10698717 PMC1220923

[jnc70533-bib-0021] Frame, L. T. , S. Ozawa , S. A. Nowell , et al. 2000. “A Simple Colorimetric Assay for Phenotyping the Major Human Thermostable Phenol Sulfotransferase (SULT1A1) Using Platelet Cytosols.” Drug Metabolism and Disposition 28, no. 9: 1063–1068. 10950850

[jnc70533-bib-0022] Gamage, N. , A. Barnett , N. Hempel , et al. 2006. “Human Sulfotransferases and Their Role in Chemical Metabolism.” Toxicological Sciences 90, no. 1: 5–22. 16322073 10.1093/toxsci/kfj061

[jnc70533-bib-0023] Ghersi‐Egea, J.‐F. , N. Strazielle , M. Catala , V. Silva‐Vargas , F. Doetsch , and B. Engelhardt . 2018. “Molecular Anatomy and Functions of the Choroidal Blood‐Cerebrospinal Fluid Barrier in Health and Disease.” Acta Neuropathologica 135: 337–361. 29368213 10.1007/s00401-018-1807-1

[jnc70533-bib-0024] Ghersi‐Egea, J.‐F. , N. Strazielle , A. Murat , A. Jouvet , A. Buénerd , and M.‐F. Belin . 2006. “Brain Protection at the Blood–Cerebrospinal Fluid Interface Involves a Glutathione‐Dependent Metabolic Barrier Mechanism.” Journal of Cerebral Blood Flow & Metabolism 26, no. 9: 1165–1175. 16395287 10.1038/sj.jcbfm.9600267

[jnc70533-bib-0025] Giordano, G. , and L. G. Costa . 2012. “Developmental Neurotoxicity: Some Old and New Issues.” International Scholarly Research Notices 2012, no. 1: 814795.

[jnc70533-bib-0026] Glatt, H. 2000. “Sulfotransferases in the Bioactivation of Xenobiotics.” Chemico‐Biological Interactions 129, no. 1–2: 141–170. 11154739 10.1016/s0009-2797(00)00202-7

[jnc70533-bib-0027] Hebbring, S. J. , A. A. Adjei , J. L. Baer , et al. 2007. “Human SULT1A1 Gene: Copy Number Differences and Functional Implications.” Human Molecular Genetics 16, no. 5: 463–470. 17189289 10.1093/hmg/ddl468

[jnc70533-bib-0028] Higaki, K. , M. Ishii , H. Esumi , M. Kanayama , K.‐I. Ogawara , and T. Kimura . 2003. “Pharmacokinetic Analysis of Factors Determining Elimination Pathways for Sulfate and Glucuronide Metabolites of Xenobiotics II: Studies With Isolated Perfused Rat Liver.” Xenobiotica 33, no. 11: 1097–1108. 14660174 10.1080/00498250310001615771

[jnc70533-bib-0029] Hirshey, S. J. , and C. N. Falany . 1990. “Purification and Characterization of Rat Liver Minoxidil Sulphotransferase.” Biochemical Journal 270, no. 3: 721–728. 2241904 10.1042/bj2700721 PMC1131791

[jnc70533-bib-0030] James, M. O. 2014. “Enzyme Kinetics of Conjugating Enzymes: PAPS Sulfotransferase.” Enzyme Kinetics in Drug Metabolism: Fundamentals and Applications 1113: 187–201. 10.1007/978-1-62703-758-7_10 24523114

[jnc70533-bib-0031] James, M. O. , and S. Ambadapadi . 2013. “Interactions of Cytosolic Sulfotransferases With Xenobiotics.” Drug Metabolism Reviews 45, no. 4: 401–414. 24188364 10.3109/03602532.2013.835613

[jnc70533-bib-0032] Klaassen, C. D. , and J. W. Boles . 1997. “The Importance of 3′‐Phosphoadenosine 5′‐Phosphosulfate (PAPS) in the Regulation of Sulfation.” FASEB Journal 11, no. 6: 404–418. 9194521 10.1096/fasebj.11.6.9194521

[jnc70533-bib-0033] Kratzer, I. , S. A. Liddelow , N. R. Saunders , K. M. Dziegielewska , N. Strazielle , and J.‐F. Ghersi‐Egea . 2013. “Developmental Changes in the Transcriptome of the Rat Choroid Plexus in Relation to Neuroprotection.” Fluids and Barriers of the CNS 10: 1–19. 23915922 10.1186/2045-8118-10-25 PMC3737068

[jnc70533-bib-0034] Kratzer, I. , N. Strazielle , E. Saudrais , et al. 2018. “Glutathione Conjugation at the Blood–CSF Barrier Efficiently Prevents Exposure of the Developing Brain Fluid Environment to Blood‐Borne Reactive Electrophilic Substances.” Journal of Neuroscience 38, no. 14: 3466–3479. 29507144 10.1523/JNEUROSCI.2967-17.2018 PMC6596044

[jnc70533-bib-0035] Kratzer, I. , A. Vasiljevic , C. Rey , et al. 2012. “Complexity and Developmental Changes in the Expression Pattern of Claudins at the Blood–CSF Barrier.” Histochemistry and Cell Biology 138: 861–879. 22886143 10.1007/s00418-012-1001-9 PMC3483103

[jnc70533-bib-0036] Lee, S. , P. A. Dawson , A. K. Hewavitharana , P. N. Shaw , and D. Markovich . 2006. “Disruption of NaS1 Sulfate Transport Function in Mice Leads to Enhanced Acetaminophen‐Induced Hepatotoxicity.” Hepatology 43, no. 6: 1241–1247. 16729303 10.1002/hep.21207

[jnc70533-bib-0037] Lehtinen, M. K. , C. S. Bjornsson , S. M. Dymecki , R. J. Gilbertson , D. M. Holtzman , and E. S. Monuki . 2013. “The Choroid Plexus and Cerebrospinal Fluid: Emerging Roles in Development, Disease, and Therapy.” Journal of Neuroscience 33, no. 45: 17553–17559. 24198345 10.1523/JNEUROSCI.3258-13.2013 PMC3818536

[jnc70533-bib-0038] Liddelow, S. A. , K. M. Dziegielewska , C. J. Ek , et al. 2013. “Mechanisms That Determine the Internal Environment of the Developing Brain: A Transcriptomic, Functional and Ultrastructural Approach.” PLoS One 8, no. 7: e65629. 23843944 10.1371/journal.pone.0065629 PMC3699566

[jnc70533-bib-0039] Liyou, N. E. , K. M. Buller , M. J. Tresillian , et al. 2003. “Localization of a Brain Sulfotransferase, SULT4A1, in the Human and Rat Brain: An Immunohistochemical Study.” Journal of Histochemistry and Cytochemistry 51, no. 12: 1655–1664. 14623933 10.1177/002215540305101209

[jnc70533-bib-0040] Löscher, W. , and H. Potschka . 2005. “Blood‐Brain Barrier Active Efflux Transporters: ATP‐Binding Cassette Gene Family.” NeuroRx 2, no. 1: 86–98. 15717060 10.1602/neurorx.2.1.86 PMC539326

[jnc70533-bib-0041] Lu, X. , Y. Song , J. Wang , et al. 2024. “Developmental Dopaminergic Signaling Modulates Neural Circuit Formation and Contributes to Autism Spectrum Disorder‐Related Phenotypes.” American Journal of Pathology 194, no. 6: 1062–1077. 38492733 10.1016/j.ajpath.2024.02.014

[jnc70533-bib-0042] Ma, B. , M. Shou , and M. L. Schrag . 2003. “Solvent Effect on cDNA‐Expressed Human Sulfotransferase (SULT) Activities In Vitro.” Drug Metabolism and Disposition 31, no. 11: 1300–1305. 14570759 10.1124/dmd.31.11.1300

[jnc70533-bib-0043] Mesia‐Vela, S. , and F. Kauffman . 2003. “Inhibition of Rat Liver Sulfotransferases SULT1A1 and SULT2A1 and Glucuronosyltransferase by Dietary Flavonoids.” Xenobiotica 33, no. 12: 1211–1220. 14742143 10.1080/00498250310001615762

[jnc70533-bib-0044] Minchin, R. F. , A. Lewis , D. Mitchell , F. F. Kadlubar , and M. E. McManus . 2008. “Sulfotransferase 4A1.” International Journal of Biochemistry & Cell Biology 40, no. 12: 2686–2691. 18248844 10.1016/j.biocel.2007.11.010

[jnc70533-bib-0045] Mizuma, T. , M. Hayashi , and S. Awazu . 1983. “P‐Nitrophenol Sulfation in Rat Liver Cytosol: Multiple Forms and Substrate Inhibition of Aryl Sulfotransferase.” Journal of Pharmacobio‐Dynamics 6, no. 11: 851–858. 6583378 10.1248/bpb1978.6.851

[jnc70533-bib-0046] Mousa, A. , and M. Bakhiet . 2013. “Role of Cytokine Signaling During Nervous System Development.” International Journal of Molecular Sciences 14, no. 7: 13931–13957. 23880850 10.3390/ijms140713931 PMC3742226

[jnc70533-bib-0047] Mulder, G. J. 1981. Sulfation of Drugs and Related Compounds. CRC Press.

[jnc70533-bib-0048] Nakamura, J. , T. Mizuma , T. Horie , M. Hayashi , and S. Awazu . 1987. “Aryl Sulfotransferase in Rat Liver: Multiplicity and Substrate Specificity.” Journal of Pharmacobio‐Dynamics 10, no. 12: 736–742. 3482862 10.1248/bpb1978.10.736

[jnc70533-bib-0049] Nishimuta, H. , H. Ohtani , M. Tsujimoto , K. Ogura , A. Hiratsuka , and Y. Sawada . 2007. “Inhibitory Effects of Various Beverages on Human Recombinant Sulfotransferase Isoforms SULT1A1 and SULT1A3.” Biopharmaceutics & Drug Disposition 28, no. 9: 491–500. 17876860 10.1002/bdd.579

[jnc70533-bib-0050] Nowell, S. , and C. Falany . 2006. “Pharmacogenetics of Human Cytosolic Sulfotransferases.” Oncogene 25, no. 11: 1673–1678. 16550167 10.1038/sj.onc.1209376

[jnc70533-bib-0051] Ozawa, S. , H. C. Chou , F. F. Kadlubar , K. Nagata , Y. Yaraazoe , and R. Kato . 1994. “Activation of 2‐Hydroxyamino‐l‐Methyl‐6‐Phenylimidazo [4, 5‐b] Pyridne by cDNA‐Expressed Human and Rat Arylsulfotransferases.” Japanese Journal of Cancer Research 85, no. 12: 1220–1228. 7852185 10.1111/j.1349-7006.1994.tb02933.x PMC5919400

[jnc70533-bib-0052] Pacifici, G. M. , M. Franchi , C. Colizzi , L. Giuliani , and A. Rane . 1988. “Sulfotransferase in Humans: Development and Tissue Distribution.” Pharmacology 36, no. 6: 411–419. 3166522 10.1159/000138330

[jnc70533-bib-0053] Percie du Sert, N. , V. Hurst , A. Ahluwalia , et al. 2020. “The ARRIVE Guidelines 2.0: Updated Guidelines for Reporting Animal Research.” Journal of Cerebral Blood Flow & Metabolism 40, no. 9: 1769–1777. 32663096 10.1177/0271678X20943823 PMC7430098

[jnc70533-bib-0054] Peterson, G. L. 1977. “A Simplification of the Protein Assay Method of Lowry et al. Which Is More Generally Applicable.” Analytical Biochemistry 83, no. 2: 346–356. 603028 10.1016/0003-2697(77)90043-4

[jnc70533-bib-0055] Qiu, F. , K. M. Dziegielewska , M. D. Habgood , Y. Huang , and N. R. Saunders . 2025. “Effects of Valproate on the Entry of Inert Hydrophilic Markers and Expression of Tight Junction Associated Genes in the Neonatal Brain and Choroid Plexus of a Rat Model of Epilepsy (GAERS).” Fluids and Barriers of the CNS 22, no. 1: 56. 40506716 10.1186/s12987-025-00667-4 PMC12160382

[jnc70533-bib-0056] Richard, K. , R. Hume , E. Kaptein , E. L. Stanley , T. J. Visser , and M. W. Coughtrie . 2001. “Sulfation of Thyroid Hormone and Dopamine During Human Development: Ontogeny of Phenol Sulfotransferases and Arylsulfatase in Liver, Lung, and Brain.” Journal of Clinical Endocrinology & Metabolism 86, no. 6: 2734–2742. 11397879 10.1210/jcem.86.6.7569

[jnc70533-bib-0057] Rivett, A. J. , A. Francis , R. Whittemore , and J. A. Roth . 1984. “Sulfate Conjugation of Dopamine in Rat Brain: Regional Distribution of Activity and Evidence for Neuronal Localization.” Journal of Neurochemistry 42, no. 5: 1444–1449. 6584547 10.1111/j.1471-4159.1984.tb02807.x

[jnc70533-bib-0058] Rochester, J. R. 2013. “Bisphenol A and Human Health: A Review of the Literature.” Reproductive Toxicology 42: 132–155. 23994667 10.1016/j.reprotox.2013.08.008

[jnc70533-bib-0059] Salman, E. D. , S. A. Kadlubar , and C. N. Falany . 2009. “Expression and Localization of Cytosolic Sulfotransferase (SULT) 1A1 and SULT1A3 in Normal Human Brain.” Drug Metabolism and Disposition 37, no. 4: 706–709. 19171676 10.1124/dmd.108.025767 PMC2680540

[jnc70533-bib-0060] Saunders, N. R. , K. M. Dziegielewska , R. M. Fame , M. K. Lehtinen , and S. A. Liddelow . 2023. “The Choroid Plexus: A Missing Link in Our Understanding of Brain Development and Function.” Physiological Reviews 103, no. 1: 919–956. 36173801 10.1152/physrev.00060.2021 PMC9678431

[jnc70533-bib-0061] Saunders, N. R. , K. M. Dziegielewska , K. Møllgård , and M. D. Habgood . 2018. “Physiology and Molecular Biology of Barrier Mechanisms in the Fetal and Neonatal Brain.” Journal of Physiology 596, no. 23: 5723–5756. 29774535 10.1113/JP275376 PMC6265560

[jnc70533-bib-0062] Saunders, N. R. , M. D. Habgood , K. Møllgård , and K. M. Dziegielewska . 2016. “The Biological Significance of Brain Barrier Mechanisms: Help or Hindrance in Drug Delivery to the Central Nervous System?” F1000Research 5: 5. 10.12688/f1000research.7378.1 PMC4786902 26998242

[jnc70533-bib-0063] Shimizu, M. , K. Ohta , Y. Matsumoto , M. Fukuoka , Y. Ohno , and S. Ozawa . 2002. “Sulfation of Bisphenol A Abolished Its Estrogenicity Based on Proliferation and Gene Expression in Human Breast Cancer MCF‐7 Cells.” Toxicology In Vitro 16, no. 5: 549–556. 12206822 10.1016/s0887-2333(02)00055-3

[jnc70533-bib-0064] Sidharthan, N. P. , R. F. Minchin , and N. J. Butcher . 2013. “Cytosolic Sulfotransferase 1A3 Is Induced by Dopamine and Protects Neuronal Cells From Dopamine Toxicity: Role of D1 Receptor‐N‐Methyl‐D‐Aspartate Receptor Coupling.” Journal of Biological Chemistry 288, no. 48: 34364–34374. 24136195 10.1074/jbc.M113.493239 PMC3843051

[jnc70533-bib-0065] Stanley, E. L. , R. Hume , and M. W. Coughtrie . 2005. “Expression Profiling of Human Fetal Cytosolic Sulfotransferases Involved in Steroid and Thyroid Hormone Metabolism and in Detoxification.” Molecular and Cellular Endocrinology 240, no. 1–2: 32–42. 16024168 10.1016/j.mce.2005.06.003

[jnc70533-bib-0066] Strazielle, N. , and J.‐F. Ghersi‐Egea . 1999. “Demonstration of a Coupled Metabolism–Efflux Process at the Choroid Plexus as a Mechanism of Brain Protection Toward Xenobiotics.” Journal of Neuroscience 19, no. 15: 6275–6289. 10414957 10.1523/JNEUROSCI.19-15-06275.1999 PMC6782833

[jnc70533-bib-0067] Strott, C. A. 2002. “Sulfonation and Molecular Action.” Endocrine Reviews 23, no. 5: 703–732. 12372849 10.1210/er.2001-0040

[jnc70533-bib-0068] Suiko, M. , Y. Sakakibara , and M.‐C. Liu . 2000. “Sulfation of Environmental Estrogen‐Like Chemicals by Human Cytosolic Sulfotransferases.” Biochemical and Biophysical Research Communications 267, no. 1: 80–84. 10623578 10.1006/bbrc.1999.1935

[jnc70533-bib-0069] Van Harreveld, A. , N. Ahmed , and D. Tanner . 1966. “Sulfate Concentrations in Cerebrospinal Fluid and Serum of Rabbits and Cats.” American Journal of Physiology‐Legacy Content 210, no. 4: 777–780. 10.1152/ajplegacy.1966.210.4.777 5906807

[jnc70533-bib-0070] Vietri, M. , C. De Santi , A. Pietrabissa , F. Mosca , and G. Pacifici . 2000. “Inhibition of Human Liver Phenol Sulfotransferase by Nonsteroidal Anti‐Inflammatory Drugs.” European Journal of Clinical Pharmacology 56, no. 1: 81–87. 10853883 10.1007/s002280050725

[jnc70533-bib-0071] Vietri, M. , A. Pietrabissa , F. Mosca , A. Rane , and G. Pacifici . 2001. “Human Adult and Foetal Liver Sulphotransferases: Inhibition by Mefenamic Acid and Salicylic Acid.” Xenobiotica 31, no. 3: 153–161. 11465392 10.1080/00498250110043481

[jnc70533-bib-0072] Visser, T. J. , E. Kaptein , H. Glatt , I. Bartsch , M. Hagen , and M. W. Coughtrie . 1998. “Characterization of Thyroid Hormone Sulfotransferases.” Chemico‐Biological Interactions 109, no. 1–3: 279–291. 9566752 10.1016/s0009-2797(97)00139-7

[jnc70533-bib-0073] Wang, L.‐Q. , and M. O. James . 2006. “Inhibition of Sulfotransferases by Xenobiotics.” Current Drug Metabolism 7, no. 1: 83–104. 16454694 10.2174/138920006774832596

[jnc70533-bib-0074] Wengle, B. 1966. “Distribution of Some Steroid Sulphokinases in Foetal Human Tissues.” European Journal of Endocrinology 52, no. 4: 607–618. 10.1530/acta.0.0520607 4223663

[jnc70533-bib-0075] Werle, E. , T. Lenz , G. Strobel , and H. Weicker . 1988. “3‐ and 4‐O‐Sulfoconjugated and Methylated Dopamine: Highly Reduced Binding Affinity to Dopamine D2 Receptors in Rat Striatal Membranes.” Naunyn‐Schmiedeberg's Archives of Pharmacology 338, no. 1: 28–34. 2853303 10.1007/BF00168808

[jnc70533-bib-0076] Wolburg, H. , and A. Lippoldt . 2002. “Tight Junctions of the Blood–Brain Barrier: Development, Composition and Regulation.” Vascular Pharmacology 38, no. 6: 323–337. 12529927 10.1016/s1537-1891(02)00200-8

[jnc70533-bib-0077] Yamamoto, A. , M.‐Y. Liu , K. Kurogi , et al. 2015. “Sulphation of Acetaminophen by the Human Cytosolic Sulfotransferases: A Systematic Analysis.” Journal of Biochemistry 158, no. 6: 497–504. 26067475 10.1093/jb/mvv062 PMC4819960

[jnc70533-bib-0078] Zhou, S.‐F. , L.‐L. Wang , Y. M. Di , et al. 2008. “Substrates and Inhibitors of Human Multidrug Resistance Associated Proteins and the Implications in Drug Development.” Current Medicinal Chemistry 15, no. 20: 1981–2039. 18691054 10.2174/092986708785132870

[jnc70533-bib-0079] Zhu, X. , M. E. Veronese , C. Bernard , L. N. Sansom , and M. McManus . 1993. “Identification of Two Human Brain Aryl Sulfotransferase cDNAs.” Biochemical and Biophysical Research Communications 195, no. 1: 120–127. 8363592 10.1006/bbrc.1993.2018

[jnc70533-bib-0080] Zhu, X. , M. E. Veronese , P. Iocco , and M. E. McManus . 1996. “cDNA Cloning and Expression of a New Form of Human Aryl Sulfotransferase.” International Journal of Biochemistry & Cell Biology 28, no. 5: 565–571. 8697101 10.1016/1357-2725(95)00164-6

