# Supplementary material for: Developmentally Regulated Expression and Activity of Sulphotransferases in the Rat Choroid Plexuses
Source: J Neurochem. 2026 Jul 27;170(7):e70533. doi: 10.1111/jnc.70533 (PMC13408200; doi:10.1111/jnc.70533)
Supplement: Supplementary file 1 — Table S1: Assessments of normality of data distribution using the Shapiro–Wilk test, including the Shapiro–Wilk test statistic (W) and corresponding p‐value for each group in either ANOVA or t‐tests. Table S2: Summary of statistical analyses including test statistics, degrees of freedom, p values and post hoc comparisons for all figures. Table S3: Expression of sulphotransferases (Counts per million, CPM) in adult rat choroid plexus and brain cortex by RNA‐seq. Table S4: Details of compounds investigated as potential substrate or inhibitors in sulphotransferase inhibition experiments, including their sources and functions, as well as chemical structures. Figure S1: Extracted ion chromatograms of PNP (138.11 m/z, upper) and PNPS (218.00 m/z, lower) in a sample from live choroid plexus sulphotransferase activity assay. [file JNC-170-e70533-s001.pdf]

Supplementary materials

Title

Developmentally regulated expression and activity of sulphotransferases in the rat choroid plexuses

Authors

Fiona Qiu[1], Nathalie Strazielle[1,2], Anne Denuziere[1], Jean-François Ghersi-Egea[1]

[1]Université Lyon-1, INSERM U1028, CNRS UMR5292, Lyon Neurosciences Research Center, Fluid Team, Bron, France

[2] Brain-i, Lyon, France

Supplementary Table 1. Assessments of normality of data distribution using the Shapiro–Wilk test, including the Shapiro–Wilk test statistic (W) and corresponding p-value for each group in either ANOVA or t tests.

A p-value of 0.05 or greater was considered consistent with a normal distribution. Note the large majority of groups satisfied the normality assumption. Although a small proportion showed statistically significant deviation, they were accepted as inspection of the Q–Q plot revealed only minor deviation from linearity, and normality tests generally have limited power with small sample sizes. In several groups, W and/or p-values could not be calculated due to sample size or identical values.

Figure	Analysis	Group	W	Normality p
**Fig. 1A**	One-way ANOVA	P1	0.8341	0.1788
		P3	0.9973	0.8998
		P8	0.8635	0.273
		P30	0.6298	0.0012
		E19	0.7967	0.1066
**Fig. 1B**	One-way ANOVA	P1	0.9332	0.6135
		P3	0.7853	0.0783
		P8	0.9621	0.7918
		P30	-	-
		E19	0.75	-
**Fig. 2**	One-way ANOVA	P3	0.9913	0.9638
		P30	0.9913	0.8213
		E19	0.8934	0.399
**Fig. 3A**	One-way ANOVA	P1	0.9584	0.7686
		P3	0.8744	0.3154
		P30	0.8757	0.3205
		E19	0.9946	0.8599
**Fig. 3B**	One-way ANOVA	P1	0.9584	0.7686
		P3	0.8744	0.3154
		P30	0.8757	0.3205
		E19	0.9946	0.8599
**Fig. 3C**	One-way ANOVA	P1	0.8885	0.3764
		P3	0.8559	0.2457
		P30	0.9156	0.5126
		E19	0.9596	0.6135
**Fig. 3D**	One-way ANOVA	P1	0.8945	0.4043
		P30	0.7789	0.0695
		E19	0.7832	0.0749

**Fig. 3E**	One-way ANOVA	P1	0.9218	0.5471
		P3	0.869	0.2939
		P30	0.9634	0.8003
		E19	0.897	0.3762
**Fig. 3F**	One-way ANOVA	P1	0.9587	0.7707
		P3	0.9792	0.8973
		P30	0.9679	0.8283
		E19	0.8721	0.3016
**Fig. 3G**	One-way ANOVA	P1	0.9784	0.8928
		P3	0.7479	0.0369
		P30	0.9091	0.4776
		E19	0.9623	0.6266
**Fig. 3H**	One-way ANOVA	P1	0.9981	0.994
		P3	0.8381	0.1899
		P30	0.7766	0.0664
		E19	0.9794	0.7251
**Fig. 4A**	One-way ANOVA	P1	0.9719	0.8531
		P3	0.9334	0.6145
		P30	0.7528	0.041
		E19	0.8746	0.3087
**Fig. 4B**	One-way ANOVA	P1	0.9721	0.8547
		P3	0.9472	0.6989
		P30	0.8946	0.4048
		E19	0.9701	0.6679
**Fig. 4C**	One-way ANOVA	P1	0.9738	0.865
		P3	0.8974	0.4183
		P30	0.9646	0.8078
		E19	0.9185	0.4472
**Fig. 4D**	One-way ANOVA	P1	0.9982	0.9944
		P3	0.9255	0.5679
		P30	0.8548	0.2422
		E19	0.8868	0.3446
**Fig. 4E**	One-way ANOVA	P1	0.9155	0.5118
		P3	0.8846	0.3587
		P30	0.7155	0.0173
		E19	0.9576	0.6038
**Fig. 4F**	One-way ANOVA	P1	0.7961	0.0954
		P3	0.9028	0.4451
		P30	0.7171	0.018
		E19	0.991	0.8182
**Fig. 6A & 7**	Mixed-effects REML	Control	-	-
		QCT	0.9634	0.6325

		AC	0.9546	0.5899
		BPA	0.9599	0.6151
		DA	0.9543	0.5884
		E2	0.9999	0.983
		T2	0.9012	0.3893
**Fig. 6B & 7**	Mixed-effects REML	Control	-	-
		QCT	0.9512	0.5745
		MFA	-	-
		AC	0.9465	0.5542
		BPA	0.7741	0.054
		DA	0.9958	0.8761
		E2	0.9211	0.4563
		T2	0.917	0.4417
**Fig. 8A**	Two-tailed unpaired T test	P1	0.9957	0.8745
		P30	0.9953	0.8691
**Fig. 8B**	Two-tailed unpaired T test	P1	0.9558	0.5955
		P30	0.8635	0.2774
**Fig. 8C**	Two-tailed unpaired T test	P1	0.771	0.047
		P30	0.9552	0.5928
**Fig. 8D**	Two-tailed unpaired T test	P1	0.8045	0.1252
		P30	0.9199	0.452
**Fig. 9A**	Two-tailed unpaired T test	2.5 uM CP	0.9957	0.8745
		0.5 uM CP	0.8435	0.2231
		2.5 uM media	0.771	0.047
		0.5 uM media	0.9999	0.9782
**Fig. 9B**	Two-tailed unpaired T test	2.5 uM CP	0.9558	0.5955
		0.5 uM CP	0.7928	0.0975
		2.5 uM media	0.8045	0.1252
		0.5 uM media	0.9994	0.9521

Supplementary Table 2. Summary of statistical analyses including test statistics, degrees of freedom, p values, and post hoc comparisons for all figures. A p-value of 0.05 or less was considered statistically significant.

Figure	Analysis	Comparison	F(DFn, DFd)	ANOVA p	Post Hoc	Adjusted p	
**Fig. 1A**	One-way ANOVA	5 age groups	F (4, 13) = 50.44	<0.0001	Tukey	P1 vs. P3	0.9988
						P1 vs. P8	<0.0001
						P1 vs. P30	<0.0001
						P1 vs. E19	0.0001
						P3 vs. P8	<0.0001
						P3 vs. P30	<0.0001
						P3 vs. E19	0.0001
						P8 vs. P30	0.0436
						P8 vs. E19	0.9124
						P30 vs. E19	0.0148
**Fig. 1B**	One-way ANOVA	5 age groups	F (4, 14) = 10.75	0.0003	Tukey	P1 vs. P3	0.9931
						P1 vs. P8	0.0141
						P1 vs. P30	0.0006
						P1 vs. E19	0.0838
						P3 vs. P8	0.0304
						P3 vs. P30	0.0013
						P3 vs. E19	0.1607
						P8 vs. P30	0.4681
						P8 vs. E19	0.9547
						P30 vs. E19	0.2143
**Fig. 2**	One-way ANOVA	3 age groups	F (2, 8) = 34.98	0.0001	Tukey	P3 vs. P30	0.199
						P3 vs. E19	0.0006
						P30 vs. E19	0.0001

**Fig. 3A**	One-way ANOVA	4 age groups	$F (3, 11) = 206.8$	<0.0001	Tukey	P1 vs. P3	0.0002
						P1 vs. P30	<0.0001
						P1 vs. E19	<0.0001
						P3 vs. P30	<0.0001
						P3 vs. E19	<0.0001
						P30 vs. E19	0.0334
**Fig. 3B**	One-way ANOVA	4 age groups	$F (3, 11) = 24.79$	<0.0001	Tukey	P1 vs. P3	0.1114
						P1 vs. P30	<0.0001
						P1 vs. E19	0.0058
						P3 vs. P30	0.0006
						P3 vs. E19	0.2526
						P30 vs. E19	0.0259
**Fig. 3C**	One-way ANOVA	4 age groups	$F (3, 11) = 18.88$	0.0001	Tukey	P1 vs. P3	0.9034
						P1 vs. P30	0.0008
						P1 vs. E19	0.7716
						P3 vs. P30	0.0003
						P3 vs. E19	0.9861
						P30 vs. E19	0.0003
**Fig. 3D**	One-way ANOVA	4 age groups	$F (3, 9) = 19.42$	0.0003	Tukey	P1 vs. P3	0.9994
						P1 vs. P30	0.9944
						P1 vs. E19	0.0004
						P3 vs. P30	0.9998
						P3 vs. E19	0.0018
						P30 vs. E19	0.0005
**Fig. 3E**	One-way ANOVA	4 age groups	$F (3, 11) = 5.181$	0.0179	Tukey	P1 vs. P3	0.3986
						P1 vs. P30	0.9286
						P1 vs. E19	0.0497

						P3 vs. P30	0.1717
						P3 vs. E19	0.4714
						P30 vs. E19	0.0195
**Fig. 3F**	One-way ANOVA	4 age groups	F (3, 11) = 1.228	0.3457	Tukey	P1 vs. P3	0.9158
						P1 vs. P30	0.3252
						P1 vs. E19	0.995
						P3 vs. P30	0.6663
						P3 vs. E19	0.9835
						P30 vs. E19	0.5104
**Fig. 3G**	One-way ANOVA	4 age groups	F (3, 11) = 5.048	0.0194	Tukey	P1 vs. P3	0.9077
						P1 vs. P30	0.1891
						P1 vs. E19	0.1
						P3 vs. P30	0.0658
						P3 vs. E19	0.0362
						P30 vs. E19	0.939
**Fig. 3H**	One-way ANOVA	4 age groups	F (3, 11) = 22.59	<0.0001	Tukey	P1 vs. P3	0.3217
						P1 vs. P30	0.0003
						P1 vs. E19	0.3782
						P3 vs. P30	0.0039
						P3 vs. E19	0.0283
						P30 vs. E19	<0.0001
**Fig. 4A**	One-way ANOVA	4 age groups	F (3, 11) = 4.000	0.0376	Tukey	P1 vs. P3	0.2684
						P1 vs. P30	0.0618
						P1 vs. E19	0.0477
						P3 vs. P30	0.782
						P3 vs. E19	0.6133
						P30 vs. E19	0.9818

**Fig. 4B**	One-way ANOVA	4 age groups	F (3, 11) = 1.295	0.7767	Tukey	P1 vs. P3	0.9975
						P1 vs. P30	0.4247
						P1 vs. E19	0.9026
						P3 vs. P30	0.3347
						P3 vs. E19	0.8275
						P30 vs. E19	0.8583
**Fig. 4C**	One-way ANOVA	4 age groups	F (3, 11) = 9.357	0.0023	Tukey	P1 vs. P3	0.3901
						P1 vs. P30	0.0914
						P1 vs. E19	0.0014
						P3 vs. P30	0.7593
						P3 vs. E19	0.0168
						P30 vs. E19	0.0771
**Fig. 4D**	One-way ANOVA	4 age groups	F (3, 11) = 1.259	0.336	Tukey	P1 vs. P3	0.9628
						P1 vs. P30	0.5728
						P1 vs. E19	0.8762
						P3 vs. P30	0.3274
						P3 vs. E19	0.6465
						P30 vs. E19	0.964
**Fig. 4E**	One-way ANOVA	4 age groups	F (3, 11) = 0.2707	0.8452	Tukey	P1 vs. P3	0.8373
						P1 vs. P30	0.9992
						P1 vs. E19	0.9833
						P3 vs. P30	0.8928
						P3 vs. E19	0.9751
						P30 vs. E19	0.9949
**Fig. 4F**	One-way ANOVA	4 age groups	F (3, 11) = 43.38	<0.0001	Tukey	P1 vs. P3	0.0891
						P1 vs. P30	<0.0001
						P1 vs. E19	0.1806

						P3 vs. P30	0.0003
						P3 vs. E19	0.0032
						P30 vs. E19	<0.0001
**Figs. 6A & 7**	Mixed-effects REML	CTRL vs 6 inhibitors	F (7, 15) = 50.09	<0.0001	Dunnett	Control vs. QCT	<0.0001
						Control vs. MFA	<0.0001
						Control vs. BPA	0.0026
						Control vs. DA	<0.0001
						Control vs. E2	0.9847
						Control vs. T2	0.8327
**Figs. 6B & 7**	Mixed-effects REML	CTRL vs 7 inhibitors	F (8, 16) = 97.12	<0.0001	Dunnett	Control vs. QCT	<0.0001
						Control vs. MFA	<0.0001
						Control vs. AC	0.001
						Control vs. BPA	<0.0001
						Control vs. DA	0.0011
						Control vs. E2	0.9939
						Control vs. T2	0.9937
**Figure**	**Analysis**	**Comparison**	**F, DFn, Dfd**	**F test p**		**p value**	
**Fig. 8A**	Two-tailed unpaired T test	2 age groups	885.7, 2, 2	0.0023		P1 vs P30	<0.0001
**Fig. 8B**	Two-tailed unpaired T test	2 age groups	56.68, 2, 2	0.0347		P1 vs P30	<0.0001
**Fig. 8C**	Two-tailed unpaired T test	2 age groups	55.41, 2, 2	0.0355		P1 vs P30	<0.0001
**Fig. 8D**	Two-tailed unpaired T test	2 age groups	27.08, 2, 2	0.0712		P1 vs P30	<0.0001
**Fig. 9A**	Two-tailed unpaired T test	2 concentrations	43.97, 2, 2	0.0445		media	0.4012
	Two-tailed unpaired T test	2 concentrations	6.553, 2, 2	0.2648		CP	0.0088
**Fig. 9B**	Two-tailed unpaired T test	2 concentrations	1.501, 2, 2	0.7998		media	0.2136
	Two-tailed unpaired T test	2 concentrations	1.699, 2, 2	0.7411		CP	0.0039

Supplementary Table 3. Expression of sulphotransferases (Counts per million, CPM) in adult rat choroid plexus and brain cortex by RNA-seq

	Choroid plexus (CPM)	Cerebral cortex (CPM)
Sult1a1	53.13	14.94
Sult1b1	0.02	0.05
Sult1c2	0.02	0.03
Sult1c2a	0.08	0.07
Sult1c3	1.48	0.03
Sult1d1	0.16	13.27
Sult1e1	0.02	0.03
Sult2a1	0.02	0.05
Sult2a2	0.02	0.03
Sult2a6	0.04	0.05
Sult2b1	0.22	9.16
Sult4a1	2.79	765.06
Sult5a1	1.19	4.91
Sult6b1	0.02	0.41

Expression of genes highlighted in red were investigated across age groups in the present study using qRT-PCR. Data from Qiu et al. (2025).

Supplementary Table 4. Details of compounds investigated as potential substrate or inhibitors in sulphotransferase inhibition experiments, including their sources and functions, as well as chemical structures

Compound	Type	Source & Function	Structure
**Quercetin**	Inhibitor	Exogenous: Flavonoid in vegetables and fruits	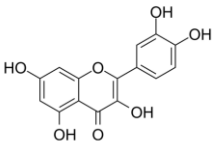
**Mefenamic acid**	Inhibitor	Exogenous: Nonsteroidal anti-inflammatory drug	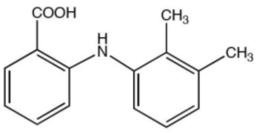
**Acetaminophen**	Substrate	Exogenous: Antipyretic and analgesic drug	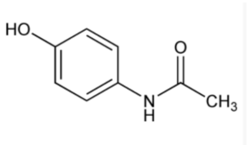
**Bisphenol-A**	Substrate	Exogenous: Environmental toxin in plastics	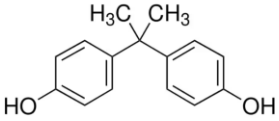
**Dopamine**	Substrate	Endogenous: Neurotransmitter	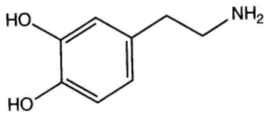
**B-estradiol**	Substrate	Endogenous: Estrogen steroid hormone	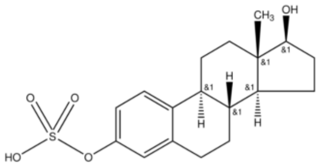
**3,5-diiodo-l-thyronine**	Substrate	Endogenous: Thyroid hormone	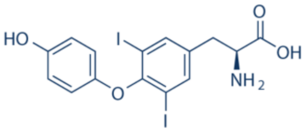

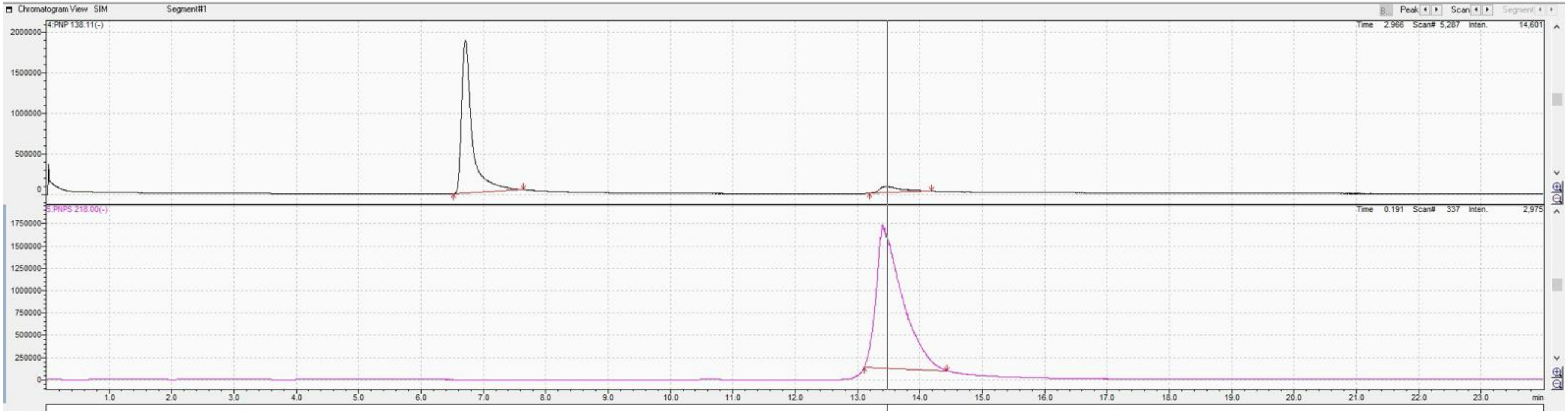

Supplementary Figure 1. Extracted ion chromatograms of PNP (138.11 m/z, upper) and PNPS (218.00 m/z, lower) in a sample from live choroid plexus sulphotransferase activity assay. The peak intensity was at arbitrary unit. Retention times were 6.7 and 13.8 minutes for PNP and PNPS respectively.
